# The mechanism underlying correlation of particulate matter-induced ferroptosis with inflammasome activation and iron accumulation in macrophages

**DOI:** 10.1038/s41420-024-01874-y

**Published:** 2024-03-15

**Authors:** Minkyung Park, Sujeong Park, Yumin Choi, Young-Lai Cho, Min Jeong Kim, Young-Jun Park, Su Wol Chung, Heedoo Lee, Seon-Jin Lee

**Affiliations:** 1 Environmental Disease Research Center, Korea Research Institute of Bioscience and Biotechnology (KRIBB), Daejeon, 34141 South Korea; 2 Department of Functional Genomics, University of Science and Technology (UST), Daejeon, 34113 South Korea; 3 Department of Biological Sciences, College of Natural Sciences, University of Ulsan, 93 Daehak-ro, Nam-gu, Ulsan, 44610 South Korea; 4 Department of Biology and Chemistry, Changwon National University, Changwon, 51140 South Korea

**Keywords:** Cell death, Immune cell death, Inflammatory diseases

## Abstract

Particulate matter (PM) is a global environmental hazard, which affects human health through free radical production, cell death induction, and immune responses. PM activates inflammasomes leading to excessive inflammatory responses and induces ferroptosis, a type of cell death. Despite ongoing research on the correlation among PM-induced ferroptosis, immune response, and inflammasomes, the underlying mechanism of this relationship has not been elucidated. In this study, we demonstrated the levels of PM-induced cell death and immune responses in murine macrophages, J774A.1 and RAW264.7, depending on the size and composition of particulate matter. PM_2.5_, with extraction ions, induced significant levels of cell death and immune responses; it induces lipid peroxidation, iron accumulation, and reactive oxygen species (ROS) production, which characterize ferroptosis. In addition, inflammasome-mediated cell death occurred owing to the excessive activation of inflammatory responses. PM-induced iron accumulation activates ferroptosis and inflammasome formation through ROS production; similar results were observed in vivo. These results suggest that the link between ferroptosis and inflammasome formation induced by PM, especially PM_2.5_ with extraction ions, is established through the iron-ROS axis. Moreover, this study can effectively facilitate the development of a new therapeutic strategy for PM-induced immune and respiratory diseases.

## Introduction

Particulate matter (PM), an air pollutant, significantly harms human health. It contains sulfates, nitrates, organic carbon, organic compounds, such as polycyclic aromatic hydrocarbons (PAHs), biological compounds, such as cell fragments or endotoxins, and heavy metals (Fe, Ca, Ni, Zn, etc.). Considering the aerodynamic diameter, they are categorized as PM with a diameter of 10 μm or less (>10 μm), PM_2.5_ (>2.5 μm), and PM_0.1_ (>0.1 μm). Moreover, based on their composition, PMs are considered metallic or organic pollutants [1]. PM including PAHs (PM PAH) contains arsenic, cadmium, nickel, lead, and several PAHs including benzo(a)anthracene, benzo(a)pyrene, benzo(b)fluoranthene, benzo(j)fluoranthene, benzo(k)fluoranthene, dibenzo(a,h)anthracene, Indeno(1,2,3-c,d)pyrene, anthracene, benzo(g,h,I)pyrene, chrysene coronene, fluoranthene, phenanthrene and pyrene [2]. PM_2.5_ with extraction ions (PM_2.5_ EI) contains organic carbon, elemental carbon, and certain water-soluble metal ions (Na^+^, K^+^, Ca^+^, Mg^2+^, Cl^-^, NO_3_^−^, and SO_4_^2−^) [3]. PM_2.5_ EI with its hazardous effects more pronounced than PM_10_ is widely recognized for its ability to penetrate into the lungs or other organs. This can give rise to several health issues, including respiratory disorders, allergic reactions, cardiovascular diseases, dermal diseases, and immune system disorders [4]. PM activates the inflammatory pathway through reactive oxygen species (ROS)-mediated mechanisms [5]. In addition, PM causes oxidative stress, lipid peroxidation, expression of inflammatory cytokine genes, and several types of cell death including ferroptosis [6, 7].

Ferroptosis is a non-apoptotic programmed cell death primarily dependent on intracellular iron overload and lipid peroxidation [8]. Moreover, Lipid peroxidation and iron accumulation, which cause ROS production leading to cell death, are key events associated with ferroptosis [9]. Glutathione peroxidase 4 (GPX4), an antioxidant enzyme that oxidizes reduced glutathione, is a major regulator of ferroptosis [8, 9]. System Xc^-^ (xCT) exchanges intracellular glutamate with extracellular cystine, the precursor of glutathione synthesis, which regulates the cysteine/glutathione ratio and protects cells from oxidative damage [8, 9]. Excess cellular iron leads to ferroptosis through the generation of ROS via the Fenton reaction [8, 9].

Inflammasomes are complexes comprising various proteins; they usually consist of a sensor protein, such as the adaptor protein NOD-like receptor family pyrin domain containing 3 (NLRP3), an apoptosis-related speckle-like protein containing the caspase recruitment domain (ASC), and the pro-inflammatory caspase caspase-1 [10]. In cells stimulated by pathogen- or damage-associated molecular patterns, intracellular ROS are generated by nicotinamide adenine dinucleotide phosphate (NADPH) oxidase or mitochondria [11]. Subsequently, activation of the inflammasome occurs, followed by autoproteolysis with activation of caspase-1, leading to degradation of IL-1β and IL-18 [10].

Despite previous reports demonstrating that PM induces inflammatory responses, inflammasome activation, iron accumulation, cellular ROS production, lipid peroxidation, and ferroptosis [7, 12], the mechanisms underlying the correlation of ferroptosis with PM-induced inflammasomes remain unclear. Therefore, we investigated different types of PM-induced cell death in macrophages, their inflammatory properties, and their correlation with inflammasomes. Using three types of PMs (PM_10_ PAHs, PM_2.5_ PAHs, and PM_2.5_ EI) to identify the most toxic components of PM, we explored the mechanisms of cytotoxicity exhibited by PMs. Understanding the mechanisms of iron- and ROS-mediated inflammasomes and ferroptosis may contribute to theoretical knowledge and aid the identification of effective therapeutic targets in inflammatory-related diseases.

## Results

### EI-containing PMs showed the most significant cytotoxic effect on macrophages

**Fig. 1:**
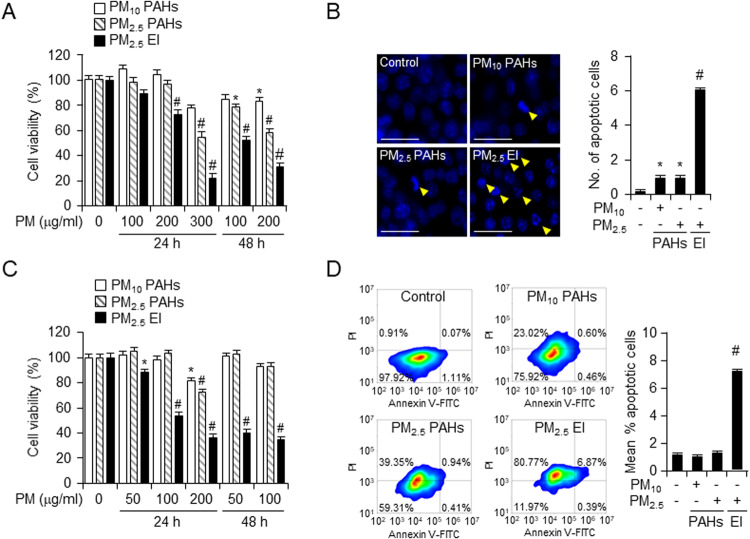
PMs induce cell death by size and component-dependent manner in macrophages. **A** Raw264.7 cells were incubated with three types of PM (100, 200 or 300 μg/ml) for 24 and 48 h. The cell viability was determined using the WST-8 assay. **B** J774A.1 cells were incubated with three types of PM (50 μg/ml) for 24 h and a Hoechst staining assay was performed using a fluorescence microscope with magnification ×40, scale bar representing 50 µm. The degree of apoptosis was evaluated and measured in eight randomly chosen microscopic fields. **C** J774A.1 cells were incubated with different concentrations of three types of PM (50, 100 or 200 μg/ml) for 24 and 48 h. The cell viability was determined using the WST-8 assay. **D** Identification of cell death in J774A.1 cell induced by PM treatment (50 μg/ml) for 24 h using Annexin-V/propidium iodide (PI) double staining followed by flow cytometry. All data are presented as the means ± standard deviations from at least three independent experiments. *P < 0.05 and #*P* < 0.001. All experiments were conducted at least three times.

We initially investigated the cytotoxicity of the three types of PM. The effect of PM on the viability of murine macrophages, RAW264.7 and J774A.1 cells, was observed using PM_10_ and PM_2.5_ containing PAHs (PM_10_ PAH_,_ PM_2.5_ PAH) and PM_2.5_ containing EIs (PM_2.5_ EI). The WST-8 assay performed using RAW264.7 and J774A.1 cells treated with 50, 100, 200, or 300 μg/ml of the three types of PM for 24 or 48 h revealed the viability of treated cells (Fig. 1A, C). Time and dose-dependent decrease in cell viability was detected after PM treatment, while, the PM_2.5_ EI treated set showed the most significant decrease. Moreover, the rate of apoptosis in PM-treated J774A.1 cells were validated to illustrate the toxicity of the three types of PMs. J774A.1 cells was exposed to 50 μg/ml of each of three PMs for 24 h. Predictably, PM_2.5_ EI induced the most frequent apoptotic cell death (Fig. 1B and D). Hence, PM_2.5_ EI might be the most cytotoxic PM among the three tested PMs, which induced cell death, including apoptosis.

### PM with EIs induced more severe inflammation than PM with PAHs

**Fig. 2:**
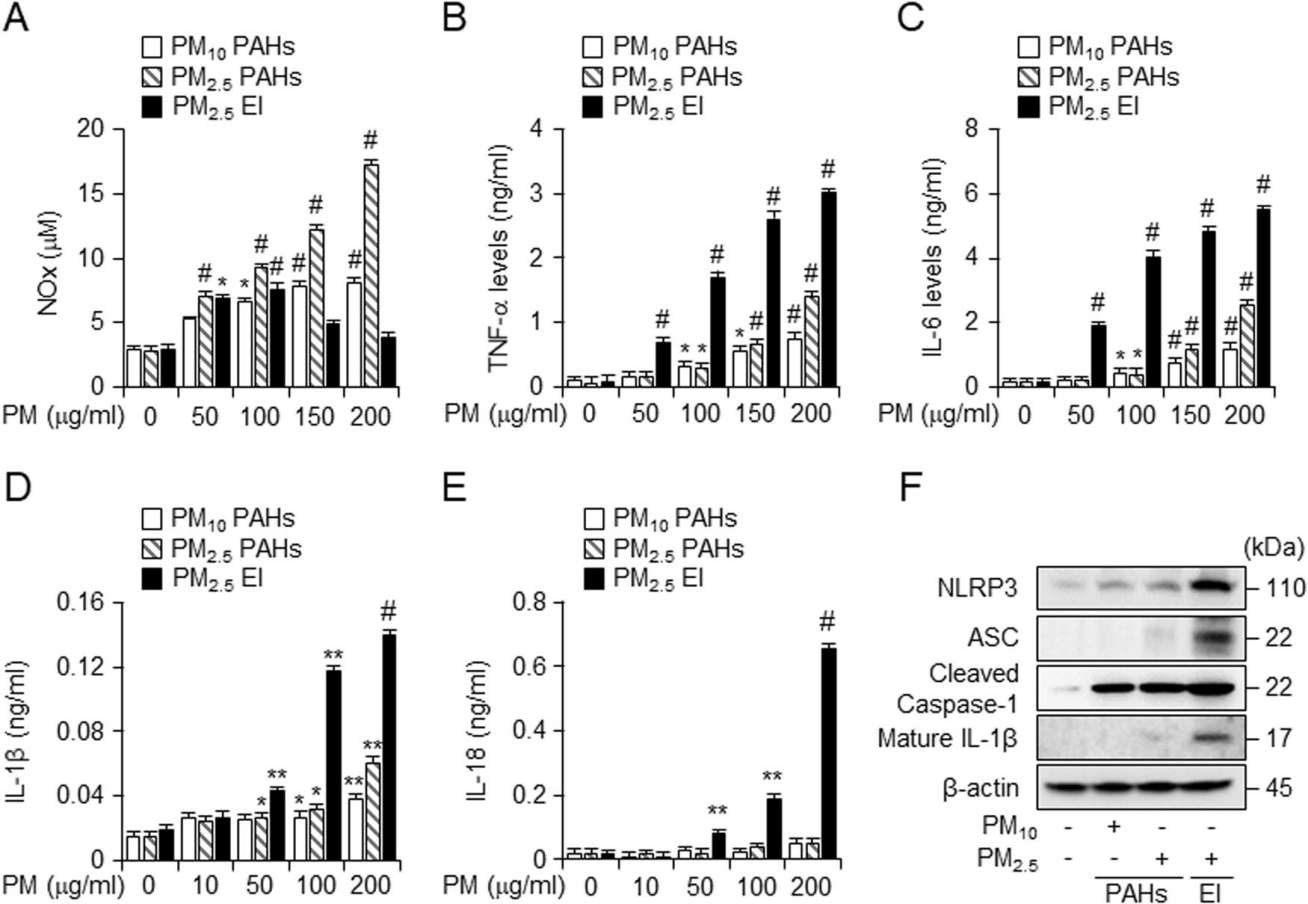
PMs increase inflammatory responses and recruit inflammasomes in macrophages. **A** J774A.1 cells were exposed to three types of PM (50, 100, 150, and 200 μg/ml) for 24 h. Amounts of nitric oxide (NO) were determined using the Griess reagent. **B**–**E** J774A.1 cells were incubated with three types of PM (10, 50, 100, 150, and 200 μg/ml) for 18 h. Effects of PM exposure on TNF-α, IL-6, IL-1β, and IL-18 levels were detected using ELISA. **F** Western blot analysis after incubation of 50 μg/ml of PM for 24 h in J774A.1 cells. All data are presented as the means ± standard deviations from at least three independent experiments. *P < 0.05, **P < 0.01 and #*P* < 0.001. All experiments were conducted at least three times.

As PM is known to induce an inflammatory response [6], we investigated whether PM exposure affects nitric oxide (NO) levels and expression of inflammatory cytokines in murine macrophage J774A.1. PM PAHs induced a dose-dependent increase in NO level. A similar pattern of increase in NO level was detected in PM_2.5_ EI-treated cells, however, it started to decrease at 150 μg/ml PM, the concentration that induced severe cell death. (Fig. 2A). ELISA revealed the effect of the three types of PM treatments on pro-inflammatory cytokines. TNF-α, IL-6, IL-1β, and IL-18 levels were assessed after exposure to 10, 50, 100, 150, or 200 μg/ml PMs for 24 h (Fig. 2B–E). All three types of PM exposure significantly increased the secretion of all pro-inflammatory cytokines in a dose-dependent manner, with PM_2.5_ EI resulting in the most enhanced levels of cytokines secretion. As inflammatory cytokines IL-1β and IL-18 increase with inflammasome activation [10], we examined the expression levels of inflammasome-associated proteins after PM exposure (50 μg/ml for 24 h) (Fig. 2F). The translation of inflammasome-related proteins increased with inflammasome activation; PM_2.5_ EI induced this change more significantly than PMs PAHs. Thus, PM_2.5_ EI could induce more severe pro-inflammation and activate inflammasomes than PM PAHs.

### PM_2.5_ EI induced ferroptosis by lipid peroxidation and iron accumulation

**Fig. 3:**
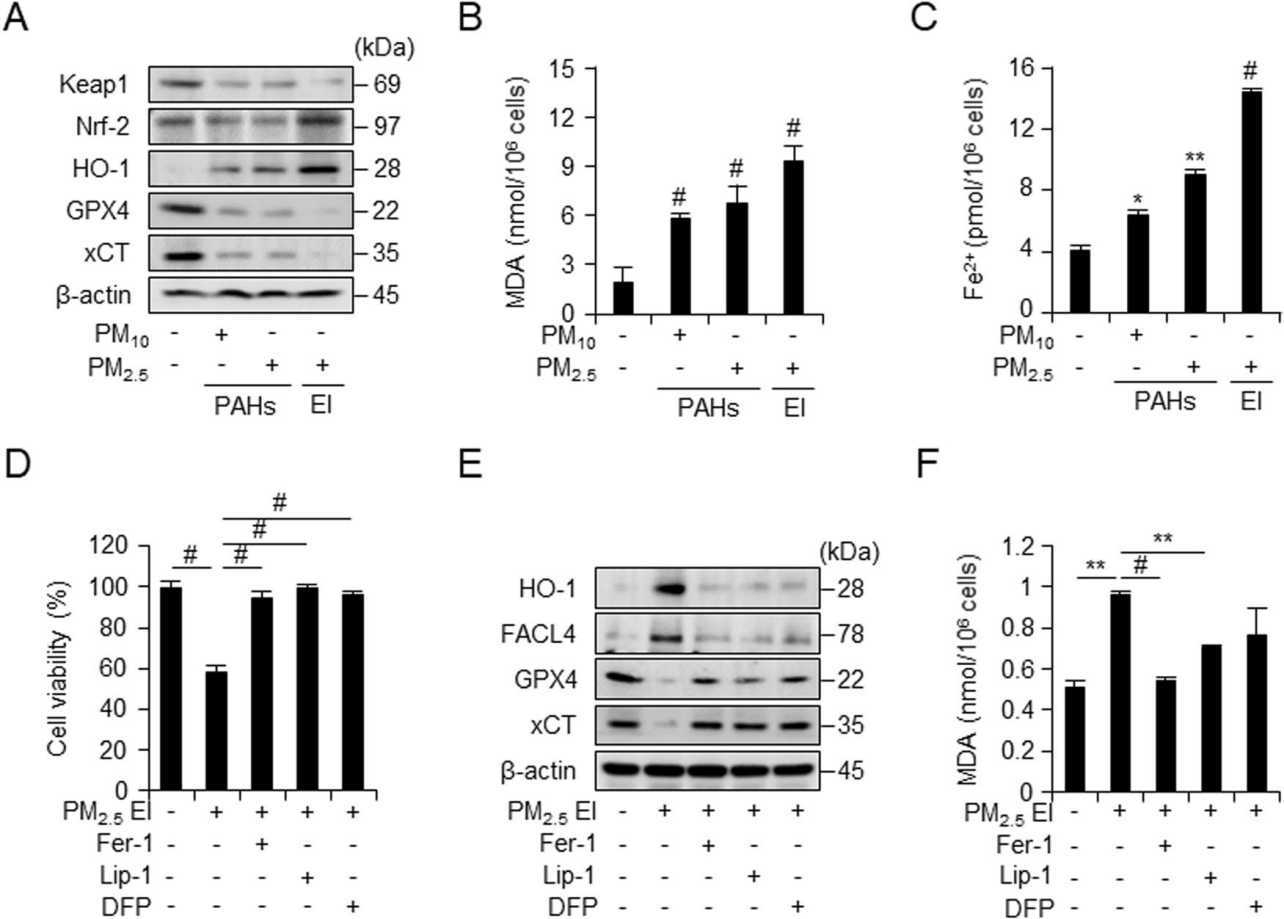
Extraction ion-containing PMs (PM_2.5_ EI) cause ferroptosis in macrophages. **A** Western blot analysis after incubation of RAW264.7 cells with three types of PM (100 μg/ml) for 18 h. **B** Malondialdehyde (MDA) formation in RAW264.7 cells after incubation with 100 μg/ml PMs for 12 h, investigated using a lipid peroxidation assay kit. **C** Intracellular ferrous iron levels are detected in J774A.1 cells incubated with 50 μg/ml of PMs for 12 h. **D** WST-8 assay demonstrating the cell viability analysis in RAW264.7 line after pretreatment with ferrostatin-1 (Fer-1; 2 μM), liproxstatin-1 (Lip-1; 2 μM), or deferiprone (DFP; 100 μM) for 2 h followed by the stimulation with 100 μg/ml of PM_2.5_ EI for 24 h. **E** Western blot analysis using RAW264.7 cells after preincubation with Fer-1 (2 μM), Lip-1 (2 μM), or DFP (100 μM) for 2 h followed by stimulation with 100 μg/ml of PM_2.5_ EI for 24 h. **F** Detection of lipid peroxidation in terms of MDA in J774A.1 cells after preincubation with Fer-1 (2 μM), Lip-1 (2 μM), or DFP (100 μM) for 2 h followed by treatment using 50 μg/ml of PM_2.5_ EI for 12 h. All data are presented as the means ± standard deviations from at least three independent experiments. *P < 0.05, **P < 0.01 and #*P* < 0.001. All experiments were conducted at least three times.

While determining the PM-induced ferroptosis in J774A.1 macrophages, we detected increased translation levels of Nrf2 and HO-1 and decreased translation levels of Keap1, GPX4, and xCT, which were associated with an increased ferroptosis, after treatment using 25 μg/ml PM for 24 h (Fig. 3A). The levels of MDA, the end product of lipid peroxidation, and intracellular ferrous iron content were measured after 25 μg/ml PM treatment for 12 h (Fig. 3B, C); the PM-induced increase in intracellular MDA and ferrous iron levels demonstrated the significant effect of PM_2.5_ EI on macrophages. To explore the association of ferroptosis with PM-induced cell death, we pretreated J774A.1 cells using the ferroptosis inhibitors ferrostatin-1 and liproxstatin-1, and the iron chelator deferiprone, followed by PM_2.5_ EI treatment. The cell viability revealed that ferroptosis inhibitors and an iron chelator prevented the PM_2.5_ EI-induced reduction in cell viability (Fig. 3D). Moreover, ferroptosis inhibitors recovered the altered translation levels of ferroptosis-related proteins regulated by PM_2.5_ EI (Fig. 3E). Similarly, MDA levels, which had been increased by PM_2.5_ EI, were suppressed by ferroptosis inhibitors and an iron chelator (Fig. 3F). The results might indicate that PM, particularly PM_2.5_ EI, induces ferroptosis in macrophages via iron accumulation and lipid peroxidation through the regulated expression of ferroptosis-related proteins.

### EI-containing PMs caused inflammasome activation in macrophages

**Fig. 4:**
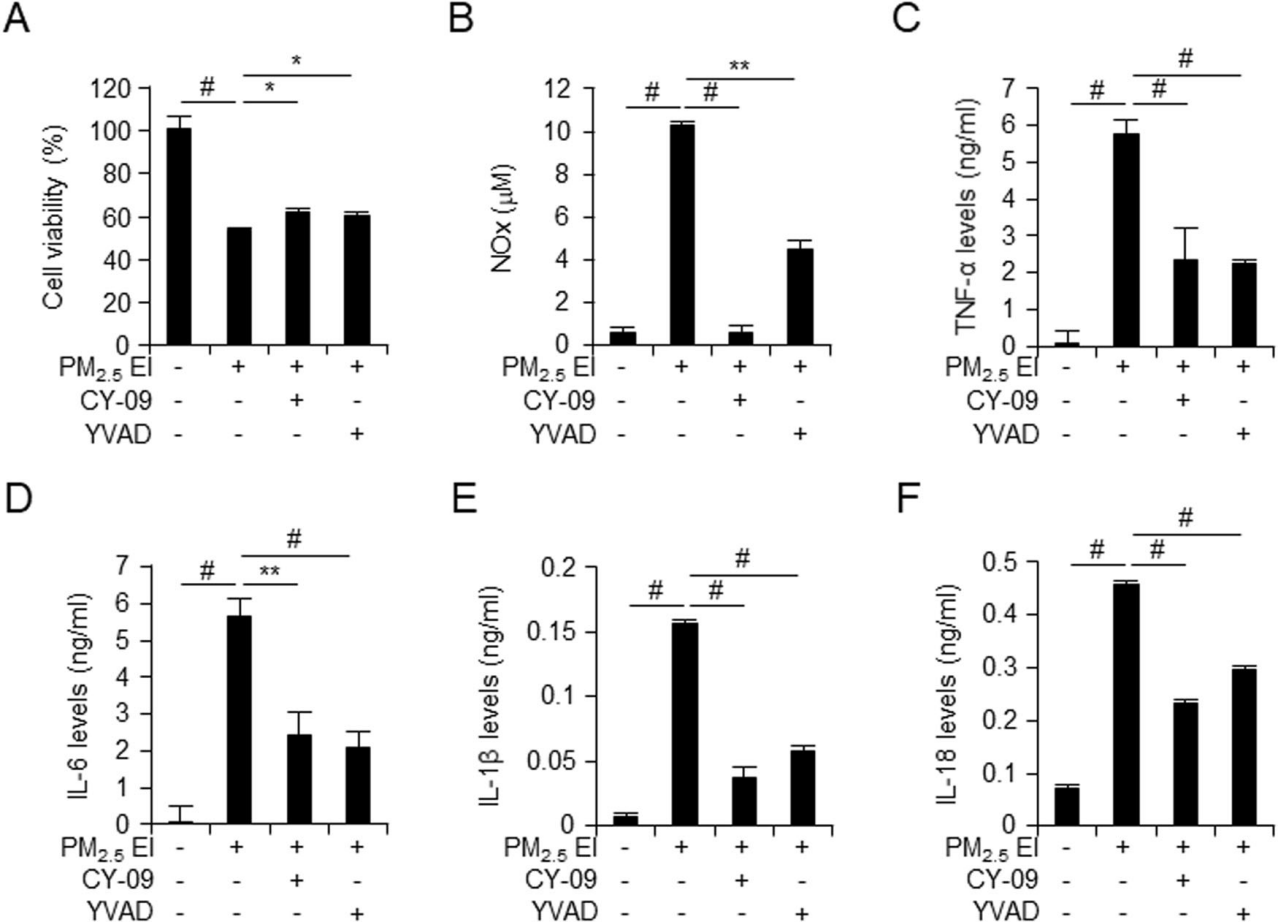
PM_2.5_ EI causes inflammasomes in macrophages. The murine macrophages J774A.1 cells preincubated with CY-09 (10 μM) or Ac-YVAD-cmk (YVAD; 20 μM) for 2 h followed by the treatment using 50 μg/ml of PM_2.5_ EI for 24 h (**A**). Cell viability detected using the WST-8 assay. **B** NO*x* levels were determined using the Griess reagent. **C**–**F** Levels of pro-inflammatory cytokines, TNF-α, IL-6, IL-1β, and IL-18, detected using ELISA. Inflammasome inhibitors such as CY-09 and Ac-YVAD-cmk attenuate nitrite levels (**B**) and secretion of pro-inflammatory cytokines as TNF-α, IL-6, IL-1β and IL-18 (**C**–**F**). All data are presented as the means ± standard deviations from at least three independent experiments. *P < 0.05, **P < 0.01 and #*P* < 0.001. All experiments were conducted at least three times.

Our results showed that particulate matters, especially PM_2.5_ EI, increased the immune response and activated the NLRP3 inflammasomes (Fig. 2). To determine whether the activation of inflammasomes mediated by PM_2.5_ EI is associated with PM_2.5_ EI-induced cell death, we examined the cell viability using 10 μM CY-09, an NLRP3 inflammasomes inhibitor, and 20 μM Ac-YVAD-cmk, a caspase-1 inhibitor, after with PM_2.5_ EI treatment (Fig. 4A). These revealed that the decreased cell viability induced by PM_2.5_ EI was recovered by treatment with CY-09 and Ac-YVAD-cmk. We also examined the induction of inflammatory and inflammasome responses in response to PM and inflammasome inhibitors. The results showed that PM-mediated increased levels of NO as well as the secretion of TNF-α and IL-6, early pro-inflammatory cytokines associated with inflammatory responses, were reduced (Fig. 4B–D). In addition, the inflammasome-induced secretion of IL-1β and IL-18, pro-inflammatory cytokines, was reduced in the presence of the inflammasome inhibitors compared to that influenced by PM alone (Fig. 4E, F). Therefore, PM may induce excessive immune responses, activate inflammasomes, and induce cell death.

### PM_2.5_ EI-mediated ferroptosis is associated with inflammasomes

**Fig. 5:**
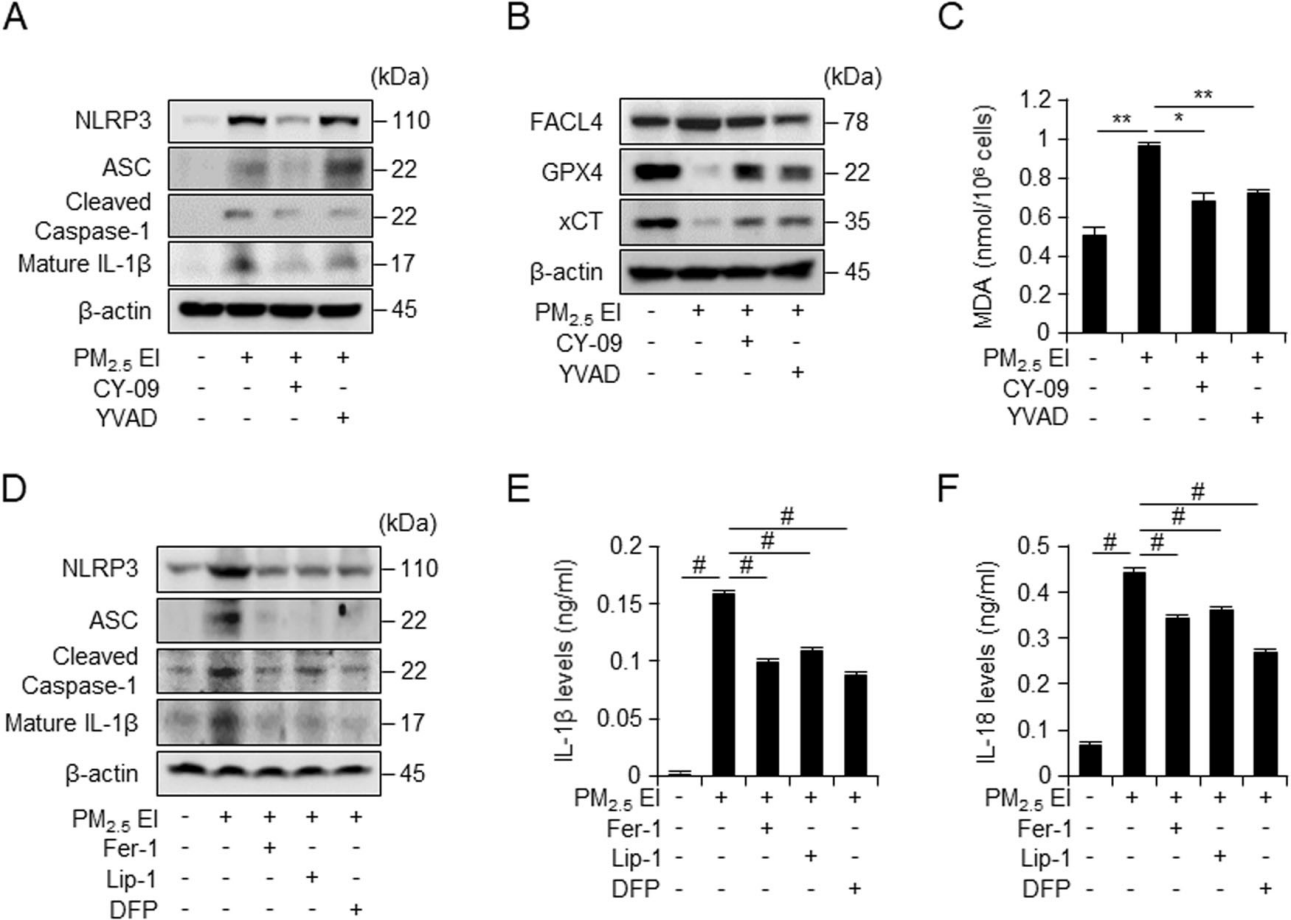
PM_2.5_ EI induces a correlation between the activation of inflammasomes and the induction of ferroptosis. **A** and **B** Western blot analysis in J774A.1 cells after preincubation with CY-09 (10 μM) or YVAD (20 μM) for 2 h and subsequent stimulation with 50 μg/ml of PM_2.5_ EI for 24 h. **C** MDA levels in J774A.1 cells after pretreatment with CY-09 (10 μM) or YVAD (20 μM) for 2 h followed by stimulation with 50 μg/ml of PM_2.5_ EI for 12 h tested using a lipid peroxidation assay kit. **D** Western blot analysis using J774A.1 cells preincubated with Fer-1 (2 μM), Lip-1 (2 μM), or DFP (100 μM) for 2 h and treated using 50 μg/ml of PM_2.5_ EI for 24 h. **E**, **F** ELISA assay revealed the IL-1β and IL-18 levels in the medium of J774A.1 cell culture after pretreatment with CY-09 (10 μM) or YVAD (20 μM) for 2 h and subsequent stimulation with 50 μg/ml of PM_2.5_ EI for 12 h. All data are presented as the means ± standard deviations from at least three independent experiments. *P < 0.05, **P < 0.01 and #*P* < 0.001. All experiments were conducted at least three times.

The western blot analysis revealed that the inflammasomes, activated by PM_2.5_ EI, was inhibited by inflammasome inhibitors in J774A.1 cell (Fig. 5A). Predictably, the expression of inflammasome-associated proteins, enhanced by PM_2.5_ EI, was reduced by the inhibitors; however, an exception of this pattern was observed in cases of NLRP3 and ASC after Ac-YVAD-cmk treatment. We observed the alteration in the expression profile of ferroptosis-related markers upon inflammasome inhibitor treatment (Fig. 5B, C). We examined the expression of FACL4, which was increased by PM_2.5_ EI exposure, and that of GPX4 and xCT, suppressed by PM_2.5_ EI exposure (Fig. 5B). Inhibition of the NLRP3 inflammasome activity suppressed ferroptosis via regulation of the translation of PM_2.5_-induced ferroptosis-related proteins. (Fig. 5B). PM_2.5_ EI enhanced MDA levels, whereas, it was suppressed by NLRP3 inflammasome inhibitors (Fig. 5C). The outcomes suggest that inhibition of inflammasomes could inhibit ferroptosis.

To determine the effect of PM-induced ferroptosis on inflammasomes, the levels of inflammasome markers were analyzed after treatment with ferroptosis inhibitors and an iron chelator. The inflammasome-related proteins, which were increased or activated by PM_2.5_ EI exposure alone, were downregulated by the treatment with ferroptosis inhibitors (Fig. 5D). We further explored the impact of ferroptosis inhibitors on the reduced secretion levels of pro-inflammatory cytokines, such as IL-1β and IL-18, which are activated by PM_2.5_ EI-induced inflammasome activation (Fig. 5E, F). Thus, these results suggest that PM_2.5_ EI-induced ferroptosis could play significant roles in the modulation of inflammasome activation, as demonstrated by the downregulation of inflammasome-related proteins following treatment with ferroptosis inhibitors and an iron chelator. The findings also could provide novel insights into the intricate interplay between PM_2.5_ EI exposure, ferroptosis, and inflammasome signaling. These results highlight a potential therapeutic avenue for mitigating PM-induced inflammatory responses.

### Inflammasomes and ferroptosis are linked by ROS generation

**Fig. 6:**
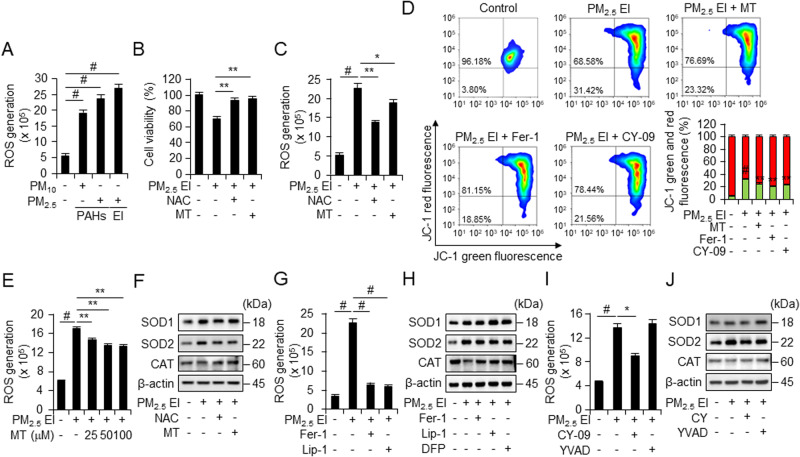
Inflammasomes and ferroptosis induced by PM_2.5_ EI are interconnected through ROS generation. **A** Detection of ROS in J774A.1 cells in response to 50 μg/ml of PM using DCFDA fluorescence staining followed by flow cytometry. **B** Cell viability analysis (WST-8 assay) in RAW264.7 after pretreatment with N-acetylcysteine (NAC; 5 mM) or mito-TEMPO (MT; 50 μM) for 2 h followed by treatment using 100 μg/ml of PM_2.5_ EI for 24 h. **C**, **G**, **I** ROS accumulation in J774A.1 cells in response to 50 μg/ml of PM_2.5_ EI treatment for 4 h following the pretreatment with NAC (5 mM), MT (50 μM), Fer-1 (2 μM), Lip-1 (2 μM), CY-09 (10 μM), or YVAD (20 μM) for 2 h tested using DCFDA fluorescence staining combined with flow cytometry. **D** Mitochondrial membrane potential destruction induced by 50 μg/ml of PM_2.5_ EI for 4 h following MT (50 μM), Fer-1 (2 μM), or CY-09 (10 μM) pre-treatment for 2 h, was evaluated through JC-1 staining followed by flow cytometry. **E** ROS generation induced by PM_2.5_ EI 4 h exposure after 2 h pre-treatment with MT was quantified using DCFDA fluorescence staining, followed by flow cytometry. **F**, **H**, **J** Western blot analysis after preincubation with NAC (5 mM), MT (10 μM), Fer-1 (2 μM), Lip-1 (2 μM), (100 μM), CY-09 (10 μM), or YVAD (20 μM) for 2 h and subsequent treatment with 50 μg/ml of PM_2.5_ EI for 4 h. All data are presented as the means ± standard deviations from at least three independent experiments. *P < 0.05, **P < 0.01 and #*P* < 0.001. All experiments were conducted at least three times.

Particulate matter induces ROS production. In this study, we investigated the role of ROS in PM_2.5_ EI-induced cell death; the most abundant ROS generation was induced by PM_2.5_ EI (25 μg/ml PM treatment for 4 h) (Fig. 6A). While exploring the protective effects of N-acetyl cysteine (NAC) and mito-TEMPO (MT) against PM-induced cell death, 5 mM NAC and 50 μM MT were detected to significantly restore cell viability after treatment using 50 μg/ml PM_2.5_ EI (Fig. 6B). Additionally, PM_2.5_ EI-mediated ROS production was suppressed by the ROS scavengers (Fig. 6C). PM_2.5_ EI also induced mitochondria membrane potential destruction, and MT, ferrostatin-1, and CY-09 showed inhibitory effects on mitochondria membrane potential destruction by PM_2.5_ EI (Fig. 6D). Moreover, MT demonstrated the dose-dependent decrease in PM-induced generation of mitochondrial ROS (Fig. 6E). Western blot analysis of PM-treated J774A.1 demonstrated that PM_2.5_ EI treatment slightly increased the expression of superoxide dismutase (SOD) 1 and 2, no significant changes in catalase (Fig. 6F). Hence, PM-induced ROS production might contributed to cell death.

Furthermore, ROS plays an important role in immune responses. We found that the PM_2.5_ EI-induced production of ROS was inhibited by ferroptosis inhibitors (Fig. 6G) and that PM_2.5_ EI-mediated ROS generation was not significantly influenced by ac-YVAD-cmk but was reduced by CY-09 (Fig. 6I). The expression levels of ROS-related proteins by PM_2.5_ EI had no significant impact by ferroptosis and inflammasome inhibitors (Fig. 6H, J). This suggests that PM_2.5_ EI-induced ROS production contributes to ferroptosis and NLRP3 inflammasome activation.

### Iron accumulation is a key factor in the correlation between ferroptosis and inflammasomes

**Fig. 7:**
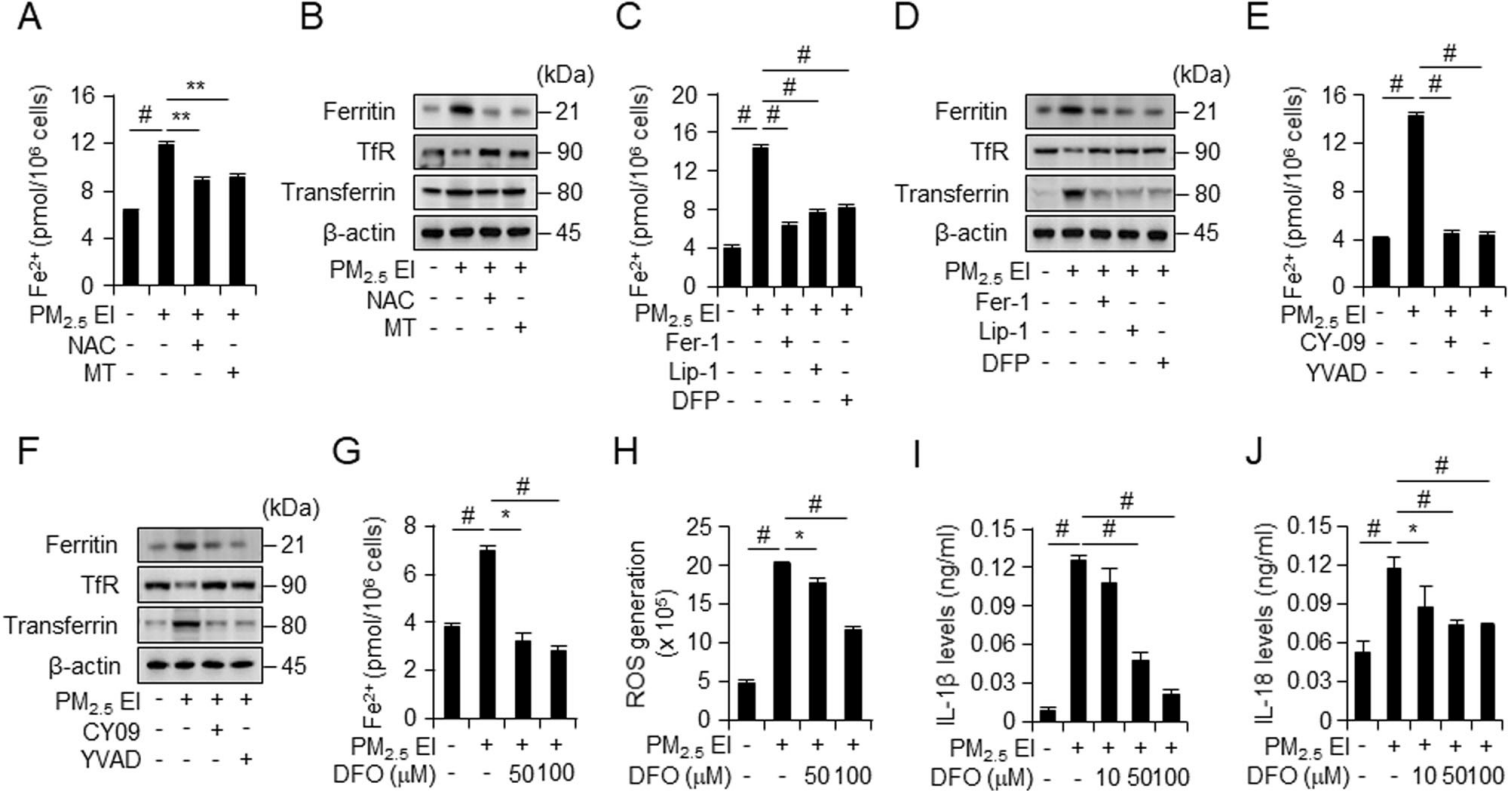
PM-induced ferroptosis and inflammasome activation are linked through the production of ROS due to iron accumulation. **A**, **C**, **E**, **G** Intracellular ferrous iron levels in J774A.1 cells after preincubation with NAC (5 mM), MT (50 μM), Fer-1 (2 μM), Lip-1 (2 μM), DFP (100 μM), CY-09 (10 μM), Ac-YVAD (20 μM), or deferoxamine (DFO; 50 and 100 μM) for 2 h followed by the treatment with 50 μg/ml of PM_2.5_ EI for 12 h detected using an iron assay kit. **B**, **D**, **F** Western blot analysis after preincubation with NAC (5 mM), MT (50 μM), Fer-1 (2 μM), Lip-1 (2 μM), DFP (100 μM), CY-09 (10 μM), or Ac-YVAD (20 μM) for 2 h and subsequent treatment with 50 μg/ml of PM_2.5_ EI for 24 h. **H** ROS levels in J774A.1 cells in response to 50 μg/ml of PM_2.5_ EI for 4 h following the pretreatment with DFO (50 and 100 μM) for 2 h detected using DCFDA fluorescence staining detected by flow cytometry. **I**, **J** IL-1β and IL-18 levels in the culture medium of J774A.1 cells after pretreatment with DFO (10, 50, and 100 μM) for 2 h and subsequent stimulation with 50 μg/ml of PM_2.5_ EI for 12 h detected using ELISA. All data are presented as the means ± standard deviations from at least three independent experiments. *P < 0.05, **P < 0.01 and #*P* < 0.001. All experiments were conducted at least three times.

The results revealed that treatment with ROS scavengers inhibited the PM_2.5_ EI-induced accumulation of ferrous iron (Fig. 7A). The expression of ferritin and transferrin, which were upregulated by PM_2.5_ EI treatment, was also inhibited by the ROS scavengers (Fig. 7B). Transferrin receptor (TfR) were not notably affected by PM_2.5_ EI (Fig. 7B). Thus, the deletion of ROS stabilized PM_2.5_ EI-induced iron accumulation, indicating that PM_2.5_ EI-induced ROS accumulation is associated with the PM_2.5_ EI-induced increase in intracellular free iron.

Increased Fe levels play important roles in ferroptosis. The treatment with ferroptosis inhibitors suppressed the PM_2.5_ EI-mediated enhancement of ferrous iron content (Fig. 7C). The expression of iron-related proteins was increased by PM_2.5_ EI; moreover, it was suppressed by ferroptosis inhibitors (Fig. 7D). We demonstrated that not only ferroptosis but also NLRP3 inflammasome inhibitors had inhibitory effects on the intracellular free iron levels and the increased expression of iron-related proteins (Fig. 7E, F). Therefore, we indicate the importance of iron in PM_2.5_ EI-induced ferroptosis and NLRP3 inflammasome activation.

Deferoxamine (DFO) is a well-known iron chelator. The levels of intracellular ferrous iron were enhanced by PM_2.5_ EI and 50 μM DFO treatment induced decreased iron levels in a dose-dependent manner (Fig. 7G). Furthermore, the treatment with DFO caused a dose-dependent decrease in PM_2.5_ EI-induced ROS production (Fig. 7H). In addition, the increase in the secretion of IL-1β and IL-18, associated with inflammasome activation, was inhibited by DFO treatment (Fig. 7I, J). Hence, iron accumulation might be crucial for ferroptosis and NLRP3 inflammasome-induced increase in ROS levels.

### Ferroptosis and inflammasomes by PM have a correlation in vivo

**Fig. 8:**
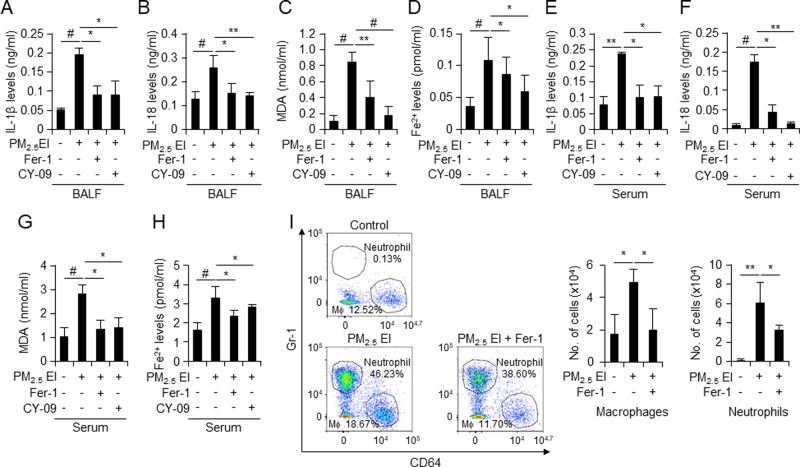
The association between PM-induced ferroptosis and inflammasomes in vivo. Fer-1 (10 mg/kg) or CY-09 (10 mg/kg) was pre-injected in mice (C57BL6/N, 6-week old, male) by intraperitoneal injection for 2 h, and PM_2.5_ EI (5 mg/kg) was subsequently administered by intratracheal instillation; after 24 h of treatment, the serum and BALFs were collected from the treated mice, and **A**, **B**, **E**, **F** serum or BALFs IL-1β and IL-18 levels were detected through ELISA. **C**, **G** MDA in serum and BALFs was detected using a lipid peroxidation assay kit. **D**, **H** Ferrous iron levels in the serum and BALFs were detected using an iron assay kit. These data are presented as the means ± standard deviations for 6 mice included in each group. **I** Analysis of macrophages and neutrophils population isolated from BALFs was detected by using flow cytometry. Macrophages and Neutrophils in BALFs were stained with anti-CD11c, anti-F4/80, anti-CD64, anti-CD11b, and anti-Gr-1 and sorted by flow cytometry. The flow cytometry data is presented with *n* = 1, and the graph displays means ± standard deviations for 6 mice included in each group. *P < 0.05, **P < 0.01 and #*P* < 0.001.

C57BL/6N 6-week-old male mice were pre-injected with ferrostatin-1 (10 mg/kg) and CY-09 (10 mg/kg) by intraperitoneal injection 2 h before the treatment of PM_2.5_ EI (5 mg/kg). Bronchoalveolar lavage fluids (BALFs) and serum of treated mice were used to observe the PM_2.5_ EI-induced levels of inflammasome and ferroptosis. ELISA assay revealed IL-1β and IL-18 levels in mouse BALF and serum; both CY-09 and ferrostatin-1 inhibited the PM-induced increase in pro-inflammatory cytokine levels (Fig. 8A, B, E, F). While investigating MDA and iron levels, markers of ferroptosis, in BALF and serum, we observed that the lipid peroxidation and iron accumulation induced by PM_2.5_ EI were inhibited by both inhibitors tested. (Fig. 8C, D, G, H). In addition, the analysis of immune cell populations in BALFs showed that the treatment with PM_2.5_ EI induced the recruitment of immune cells into the lungs, resulting in an observed increase in the numbers of macrophages and neutrophils compared to the control group (Fig. 8I). Also, we showed that ferrostatin-1 inhibited the enhanced recruitment of immune cells induced by PM_2.5_ EI (Fig. 8I). Thus, a correlation between PM_2.5_ EI-induced inflammasome activation and ferroptosis was elucidated in vivo.

## Discussion

In this study, we demonstrated that extraction ion components of particulate matter, rather than PAH constituents, exert a significantly more pronounced lethal impact on immune cells. PM with extraction ions (PM_2.5_ EI) triggered excessive immune responses in macrophages, leading to the activation of inflammasomes along with associated cell death mechanisms such as pyroptosis and also induced ferroptosis. These cellular responses were inhibited by suppressing the underlying causes of ROS, including the inhibition of iron accumulation, lipid peroxidation, and also reducing ROS production. Our findings might provide fresh insight into the genesis of immunological disorders triggered by PM, especially including the extraction of metal ions.

PM exposure induces various types of cell death including apoptosis, autophagy, necrosis, pyroptosis, and ferroptosis [6]. We investigated the effects of three types of PM (PM_10_ PAHs, PM_2.5_ PAHs, and PM_2.5_ EIs) on macrophage cytotoxicity and demonstrated that PM-induced ferroptotic cell death by increasing lipid peroxidation and iron accumulation via regulated expression of ferroptosis-related proteins. In addition, PM_2.5_ EIs more significantly induced cell death and ferroptosis than PM PAHs.

Inflammation is critically involved in many PM-associated diseases [13]. High concentrations of PM_2.5_ EI could not increase NO production more than that induced by lower concentrations, but increased secretion of TNF-α, IL-6, IL-1β, and IL-18, the pro-inflammatory cytokines. This indicates that PMs induced inflammation, however, NO production by excessive PM_2.5_ EI was interrupted owing to cell death. Activation of inflammasomes leads to the secretion of IL-1β and IL-18, triggering inflammatory responses. Excessive activation of inflammasomes and inflammatory responses can lead to pyroptosis [14]. PM induces respiratory inflammation associated with pulmonary disease, lung fibrosis, and cardiac disease through inflammasome activation [15–17]. We showed that PMs enhanced translational levels of NLRP3 and ASC, which constitute the inflammasome, and activated caspase-1 and IL-1β, which indicates that PM, especially PM_2.5_ EI, induces inflammasome activation via excessive inflammatory responses.

In this study, we aimed to determine the correlation between inflammasomes and PM-induced ferroptosis. Recently, the association of inflammasomes with ferroptosis and lipid peroxidation has been illustrated [18]. Our results detected that inhibitors of inflammasomes suppressed ferroptosis, and inhibitors of ferroptosis and iron chelator suppressed inflammasome formation. FACL4 promotes lipid peroxidation, and thus, plays a critical role in ferroptosis [19]. Activated NLRP3 and FACL4 colocalize to the mitochondrial endoplasmic reticulum [19, 20], and the NLRP3 inflammasome inhibitor CY-09 inhibits FACL4 [21], which is consistent with our results demonstrating that CY-09 and Ac-YVAD-cmk (caspase-1 inhibitor) downregulated FACL4 and inhibited PM_2.5_ EI-induced upregulation of lipid peroxidation. Therefore, the PM_2.5_ EI-induced NLRP3 inflammasome and ferroptosis possibly interact by FACL4.

The PM, we used, contains diverse metal ions and these increase cellular iron levels [3, 22]. PM exposure increases ROS levels, resulting in lipid peroxidation, and ferroptotic cell death [3, 6]. ROS from NDAPH oxidase, mitochondria, the Fenton reaction, or activation of NLRP3 initiate inflammasomes activation [11, 23, 24]. We revealed that PM_2.5_ EI induced ROS generation but there’s no significant effect on the expressions of ROS-related proteins. This suggests that the ROS induced by PM_2.5_ EI is maybe generated due to factors other than the regulation of related protein expression. These may include the elevation of intracellular free iron levels caused by PM_2.5_ EI. Ferroptosis and NLRP3 inflammasome activation is mediated iron-driven ROS-associated oxidative cell death via the Fenton reaction that leads to lipid peroxidation [24, 25]. Additionally, PM_2.5_ EI exposure upregulated ferritin, an intracellular iron storage protein, which exhibits a protective effect on ferroptosis by regulating intracellular iron levels [26]. Ferritin is involved in inflammation and inflammasome activation through regulation by pro-inflammatory cytokines, such as TNF-α, IL-6, and IL-1β and increased expression of NLRP3, caspase-1 activation, and IL-1β secretion [27–29]. PM_2.5_ EI-induced increase in ferritin levels may activate NLRP3 inflammation, which can affect the induction of ferroptosis via ROS generation. Thus, water soluble metal ions in PM_2.5_ EI might induce cellular iron accumulation, generate excessive ROS, and induce inflammasome activation and ferroptosis.

We confirmed the interplay between PM_2.5_ EI, ferroptosis and inflammasome. ROS generation increases the secretion of inflammatory cytokines and inflammatory cytokines also can generate ROS [30–32], and our study not only demonstrates an actual increase in ROS levels upon PM_2.5_ EI treatment but also reveals an elevation in inflammatory cytokine production. The increase in inflammatory cytokines could induce ferroptosis, and conversely, ferroptosis forms a positive feedback relationship that activates the secretion of inflammatory cytokines [33–35]. We verified ferroptosis and inflammasome inhibitors effectively suppressed their respective processes. Consequently, the increased levels of inflammatory cytokines through this process, might further enhance ferroptosis. Hence, the heightened immune response due to ferroptosis may trigger an excessive immune reaction, inducing inflammasome activation and pyroptosis. Simultaneously, the increased inflammatory cytokines from inflammasome activation could contribute to the induction of ferroptosis. Therefore, ferroptosis and inflammasome establish a positive feedback loop, influencing each other.

When mitochondrial function is compromised, not only does NF-κB signaling become activated, but it also induces NLRP3 inflammasome activation, leading to an increase in the secretion of pro-inflammatory cytokines [36]. Additionally, mitochondrial dysfunction can trigger ferroptosis through an increase in oxidative stress, and conversely, ferroptosis can cause changes such as mitochondrial swelling, cristae reduction or disappearance, and alterations in mitochondrial membrane potential and permeability [37, 38]. We observed that exposure to PM_2.5_ EI resulted in mitochondria membrane potential disruption, accompanied by an increase in mitochondrial reactive oxygen species, mtROS. This might underscore the significance of mitochondrial dysfunction in cell death processes, including immune responses, pyroptosis, and ferroptosis, triggered by PM_2.5_ EI exposure. Therefore, further research aimed at restoring mitochondrial function may hold potential for treating diseases caused by exposure to PMs.

Exposure to PM_2.5_ EI increased levels of MDA and iron levels related to ferroptosis and inflammatory cytokines, inflammasome-related markers, in both serum and BALF. Treatment with ferroptosis and inflammasome inhibitors reversed these PM_2.5_ EI-induced effects. Additionally, the recruitment of immune cells in mouse lungs triggered by PM_2.5_ EI was inhibited by a ferroptosis inhibitor. These findings could suggest that PM not only locally affects inflammation and ferroptosis but also has systemic consequences. Using inhibitors for ferroptosis and inflammasome may hold promise for treating diseases caused by PMs.

The finding of this study underscore the multifaceted impact of air pollution, particularly particulate matter, on cellular health. The interplay between ferroptosis and inflammasome activation in response to PM exposure is a novel and significant discovery. It suggests that the health consequences of air pollution extend beyond respiratory issues and may encompass systemic effects through inflammation and cell death processes. Furthermore, the identification of specific inhibitors that can modulate these pathways presents potential therapeutic opportunities. Developing interventions that target inflammasome activation and ferroptosis could be beneficial for mitigating the adverse health effects of air pollution. In conclusion, this study contributes to our understanding of the complex mechanisms through which PM exposure affects human health. It highlights the need for continued research into air pollution’s health impacts and potential therapeutic strategies to mitigate its effects on individuals and communities. This knowledge is essential in the ongoing efforts to address environmental and public health challenges associated with air pollution.

## Materials and methods

### Animals

Male C57BL/6N mice (6-week-old) were purchased from Orient Bio (Seongnam, South Korea). The mice were randomly divided into 4 groups (*n* = 6): control group, PM_2.5_ EI group (5 mg/kg), Ferrostatin-1 group (10 mg/kg), and CY-09 group (10 mg/kg). Ferrostatin-1 and CY-09 were intraperitoneally injected two hours prior to the intratracheal administration of PM_2.5_ EI. BALFs and serum were collected from the treated mice 24 h post PM treatment, for further assessments, such as ELISA assay, the detection of iron or MDA, and the analysis of the immune cells population. The mice were housed in the environment with temperatures maintained at 22 ± 2 °C, following a 12 h light and 12 h dark cycle, and with a humidity level of 50 ± 10%. Food and water were available without restrictions. The mice maintained following the guidelines and approval of the Institutional Review Committee for Animal Care and Use (Korea Research Institute of Bioscience and Biotechnology, KRIBB, KRIBB-AEC-233341).

### Cell culture

The murine macrophage cell lines, Raw264.7 and J774A.1, were obtained from the Korean Cell Line Bank (KCLB, Seoul, Korea). The Raw264.7 cells were cultured using Dulbecco’s modified Eagle’s medium (SH30243.01; Hyclone, Logan, UT, USA) supplemented with 10% calf serum (26170-043; GibcoTM, Waltham, MA, USA) and 1% antibiotic (LS 203-01; WELGENE Inc., Gyeongsan, Korea). The J774.A1 cells were cultured using Dulbecco’s modified Eagle’s medium (SH30243.01; Hyclone, Logan, UT, USA) supplemented with 10% fetal bovine serum (SH30919.03; Hyclone, Logan, UT, USA), 1% antibiotic (LS 203-01; WELGENE Inc., Gyeongsan, Korea), and 25 mM HEPES (15630-080; GibcoTM, Waltham, MA, USA) at 37 °C under controlled environment with 5% CO_2_ in a humidified incubator.

### Reagents and antibodies

PMs (PM_10_ and PM_2.5_) were purchased from European Reference Materials (ERM-CZ100 and ERM-CZ110; B-2440, Geel, Belgium). PM_2.5_ PAH was extracted from PM_10_-certified reference material (ERM-CZ100) using a modified sedimentation method [39]. Ferrostatin-1 (SML0583), liproxstatin-1 (SML1414), deferiprone (379409), and deferoxamine (D9533) were purchased from Sigma-Aldrich (St. Louis, MO, USA). CY-09 (HY-103666) was supplied by MedChemExpress (Monmouth Junction, NJ, USA). Ac-YVAD-CHO (10016) and mito-tempo (16621) were purchased from Cayman Chemicals (MI, USA). N-acetyl-L-cysteine (A0905) was purchased from Tokyo Chemical Industry Co. (Tokyo, Japan). The following antibodies were used: β-actin (sc-47778), SOD1 (sc-11407), SOD2 (sc-30080), catalase (sc-50508), and Keap1 (sc-365626) supplied by Santa Cruz Biotechnology Inc. (Santa Cruz, CA, USA), NLRP3 (15101T), ASC (67824T), cleaved caspase-1 (89332T), cleaved-IL-1beta (63124T), Nrf-2 (12721S), GPX4 (52455S), and xCT (12691S) provided by Cell Signaling Technology Co. (Danvers, MA, USA), HO-1 (ADI-SPA-816) obtained from (Enzo Life Sciences, Inc., Farmingdale, NY, USA), FACL4 (NBP2-16401) supplied by Novus Biologicals (Littleton, CO, USA), ferritin (ab75973) and transferrin (ab1223) provide by Abcam (Cambridge, MA, USA), and transferrin receptor (13-6800) provided by Thermo Scientific (Rockford, IL, USA).

### Cell viability assay

Cell viability was assessed using a WST-8 assay (QM10000; Biomax, Seoul, Korea). For this purpose, Raw264.7 and J774A.1 cells were plated in 96 well plates (5 × 10^4^ cells/well) for 24 h. The cells were incubated with or without different concentrations of PM_10_ PAHs, PM_2.5_ PAHs and PM_2.5_ EI (RAW264.7 cells: 100, 200, 300 μg/ml; J774A.1 cells: 50, 100, or 200 μg/ml) for 24 or 48 h. WST-8 (10 μl) was then added to each well and incubated at 37 °C for 30 min to 1 h in the dark; subsequently, the absorbance of the sample was measured at 450 nm using a SpectraMax ABS Plus microplate reader (Molecular Devices, San Jose, CA, USA).

### Hoechst 33342 staining

For detecting apoptosis in the J774A.1 line, cells were seeded on the confocal dish (211350, SPL, Pocheon, South Korea) and treated with three types of PM (100 μg/ml). The cells were washed with 1x PBS 24 h after treatment and incubated with 1 μg/ml Hoechst 33342 solution (62249; Thermo Scientific, IL, USA) for 10 min at 37 °C. Apoptosis was detected through fluorescence microscopy (Olympus, BX63, NY, USA). The extent of apoptosis was evaluated and measured in eight randomly chosen microscopic fields.

### Quantification of apoptosis by flow cytometry

The level of apoptosis was assessed using FITC Annexin V and PI (FITC Annexin V/Dead Cell Apoptosis Kit for Flow Cytometry; V13242, Invitrogen, Carlsbad, CA, USA) following the instructions provided by the manufacturer. Samples were analyzed using a NovoCyte Flow Cytometer (150014; Agilent Technologies, Inc., Santa Clara, CA, USA).

### Measurement of levels of TNF-α, IL-6, IL-1beta, IL-18 and NO metabolites

The levels of nitrite, a stable oxidized product of NO, were measured in the culture media using the Griess reagent. Samples were incubated with equal volumes of sulfanilamide and N-(1-Naphthyl) ethylenediamine solution for 5–10 min. Subsequently, absorbance was measured at 550 nm using a SpectraMax ABS Plus microplate reader (Molecular Devices). The levels of TNF-α, IL-6, IL-1 beta, and IL-18 in the culture medium were measured using the Duoset ELISA system (DY410, DY406, DY401, DY7625-05; R&D Systems, Minneapolis, MN, USA) as per the manufacturer’s instructions.

### Western blot analysis

The cells were lysed on ice by treating them with radio-immunoprecipitation assay lysis buffer and 1x protease inhibitor cocktail (Sigma-Aldrich, St Louis, MO, USA) for 30 min. The lysates were quantified using the Pierce BCA Protein Assay kit (23209; Thermo Scientific, Rockford, IL, USA). Sodium dodecyl sulfate-polyacrylamide gel electrophoresis was performed to separate 10–40 μg of proteins using a 10–15% gel, which was subsequently transferred to a polyvinylidene difluoride membrane using a Trans-Blot® TurboTM Transfer pack (Bio-Rad, CA, USA). The membranes were blocked using 5% skim milk dissolved in phosphate-buffered saline with 1% Tween 20 (PBST) for 1 h and incubated with primary antibodies overnight at 4 °C. After washing the membrane three times with PBST, they were incubated with horseradish peroxidase (HRP)-conjugated secondary antibodies for 1 h at room temperature. Next, after washing for 2 h with PBST, the protein bands were visualized using Clarity Western ECL Substrate (1705061; Bio-Rad, CA, USA).

### Detection of malondialdehyde (MDA)

Lipid peroxidation was detected by quantifying the MDA concentration in cell lysates, mice serum and BALFs using a lipid peroxidation (MDA) Assay Kit (ab118970, Abcam, CA, UK) according to the protocol provided by the manufacturer.

### The measurement of iron accumulation

The level of ferrous iron was assessed by quantifying the iron concentration in cell lysates, mice serum and BALFs using an Iron Assay Kit (colorimetric) (ab83366, Abcam, CA, UK) according to the manufacturer’s protocol.

### Detection of ROS generation

In J774A.1 cells, ROS formation was detected using a DCFDA/H2DCFDA-Cellular ROS Assay Kit (ab113851, Abcam, CA, UK) according to the manufacturer’s protocol. Briefly, cells were plated in six-well plates and pre-treated with various inhibitors for 2 h; subsequently, they were incubated with or without PMs for 4 h. The treated cells were washed with cold 1x PBS and harvested using cell lifters. For staining the cells, they were incubated with 20 μM 2′,7′-dichlorofluorescin diacetate (DCFDA) for 20 min and analyzed using NovoCyte Flow Cytometer (150014, Agilent Technologies, Inc., CA, USA).

### Detection of mitochondrial membrane potential

Mitochondrial membrane potential in J774A.1 cell was assessed using fluorescent dye JC-1 (5,5′, 6,6′-tetrachloro-1,1′, 3,3′-tetraethyl tetrethyl benzimidalyl carbocyanine iodide) MitoMP Detection Kit (MT09, Dojindo, Kumamoto, Japan) according to the manufacturer’s protocol. Briefly, J774A.1 cells treated with or without inhibitors in the presence of PM_2.5_ EI. After treatment, J774A.1 cells were harvested via lifter and stained with 2 μM JC-1 for 30 min at 37 °C after treatment with PM_2.5_ EI. JC-1 fluorescence was detected using NovoCyte Flow Cytometer (150014, Agilent Technologies, Inc., CA, USA).

### Isolation of immune cells in BALF

BALF samples were centrifuged at 500 × *g* for 5 min at 4 °C and added RBC lysis buffer (130-094-183, Miltenyi Biotec, Seoul, Korea) and incubated for 3 min at 37 °C. 1x PBS was added and centrifuged at 215 × *g* for 5 min at 4 °C and counted the number of cells. The additional centrifuge was performed at 215 × *g* for 5 min at 4 °C. Warmed fixation buffer (420801, BioLegend, CA, USA) was added and incubated for 10 min at room temperature in the dark. BAL cells were washed with FACS buffer (PBS 50 ml + FBS 1 ml + 0.5 M EDTA 20 μl) and an extra centrifugation step was carried out at 215 × *g* for 5 min at 4 °C.

### Analysis of immune cell populations using flow cytometry analysis

BAL cells were resuspended in FACS buffer and centrifuged 215 × *g* for 5 min at 4 °C. BAL cells were stained for 15 min at 4 °C in the dark with the following antibodies used per manufacturer’s instructions The following antibodies were used for flow cytometry analysis (dilutions were 1:100): APC/Cyanine7 anti-mouse CD11c Antibody (117324), PE anti-mouse F4/80 Antibody (123110), Alexa Fluor® 647 anti-mouse CD64 (FcγRI) Antibody (139322), Alexa Fluor® 488 anti-mouse/human CD11b Antibody (101217), and PerCP anti-mouse Ly-6G/Ly-6C (Gr-1) Antibody (108426), which were purchased from BioLegend, San Diego, CA, USA. Cells were gated on lymphocytes or macrophage-sized, singlet, live cells. Flow cytometry analysis was performed using a NovoCyte Flow Cytometer (150014, Agilent Technologies, Inc., CA, USA).

### Statistical analysis

Quantitative data are presented as the mean ± standard deviation. The significance was determined by performing a two-tailed, unpaired Student’s *t*-test or two-way ANOVA followed by Dunn’s post-test. Statistical significance was set at *P < 0.05, **P < 0.01, and #*P* < 0.001. We included statistical values for every experiment.

### Supplementary information

Original Data File

## Data Availability

All data generated or analyzed during this study are included in this published article, and available from the corresponding author on reasonable request.
