# Supplementary material for: The mechanism underlying correlation of particulate matter-induced ferroptosis with inflammasome activation and iron accumulation in macrophages
Source: Cell Death Discov. 2024 Mar 15;10:144. doi: 10.1038/s41420-024-01874-y (PMC10943117; doi:10.1038/s41420-024-01874-y)
Supplement: Supplementary file 1 — Original Data File [file 41420_2024_1874_MOESM1_ESM.pptx]

## Slide 1
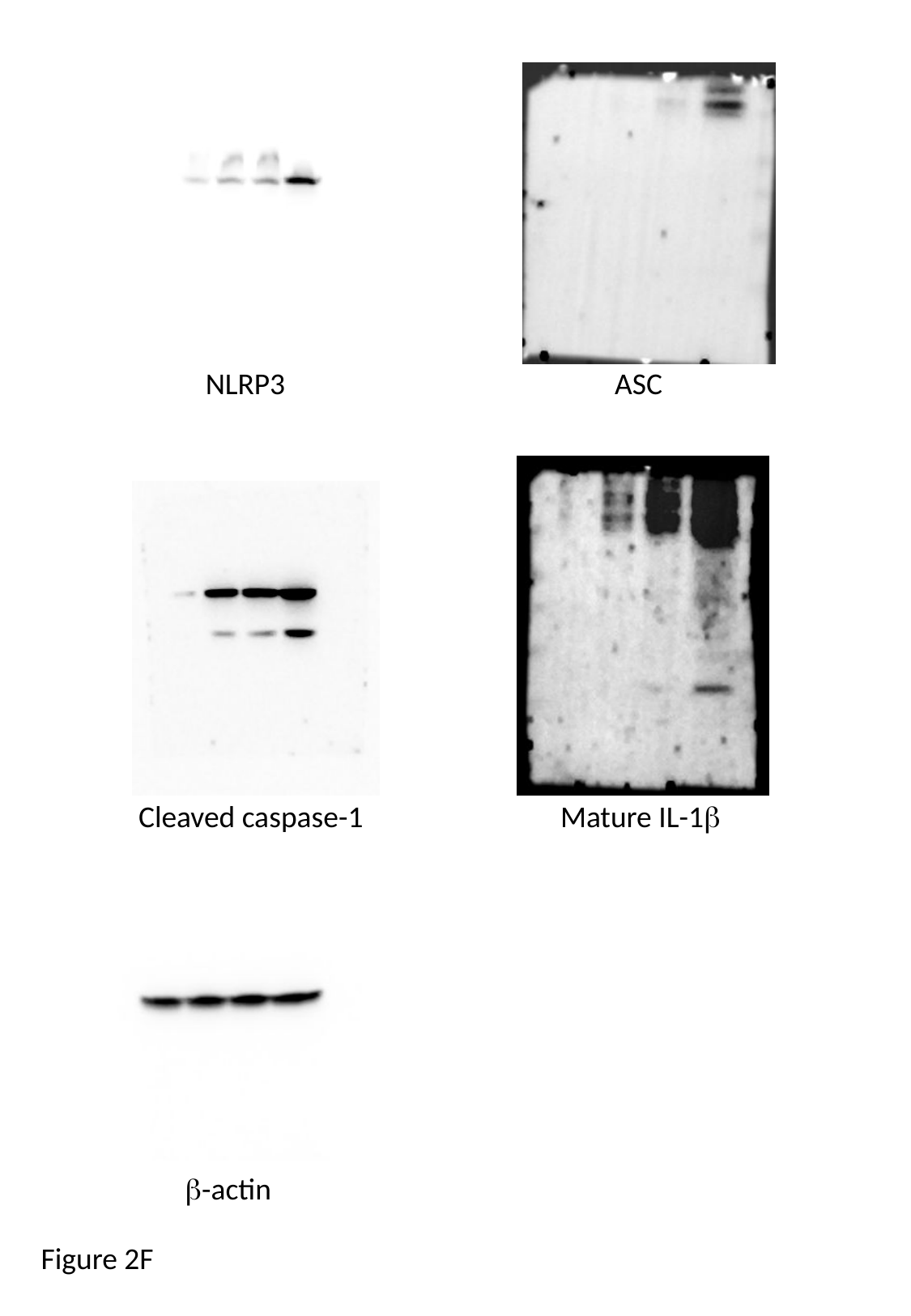

NLRP3
ASC
Cleaved caspase-1
Mature IL-1β
β-actin
Figure 2F

## Slide 2
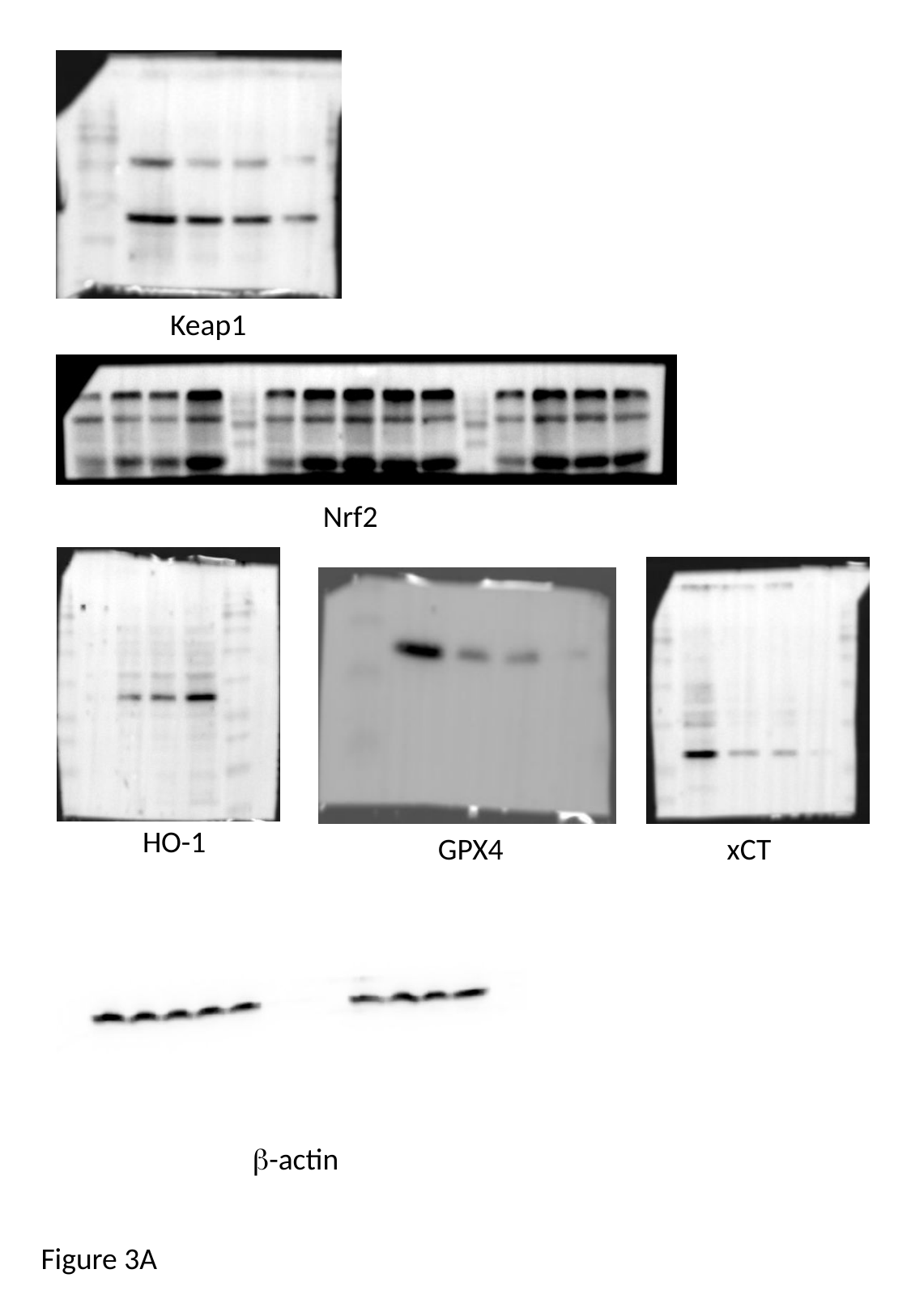

Keap1
Nrf2
HO-1
GPX4
xCT
β-actin
Figure 3A

## Slide 3
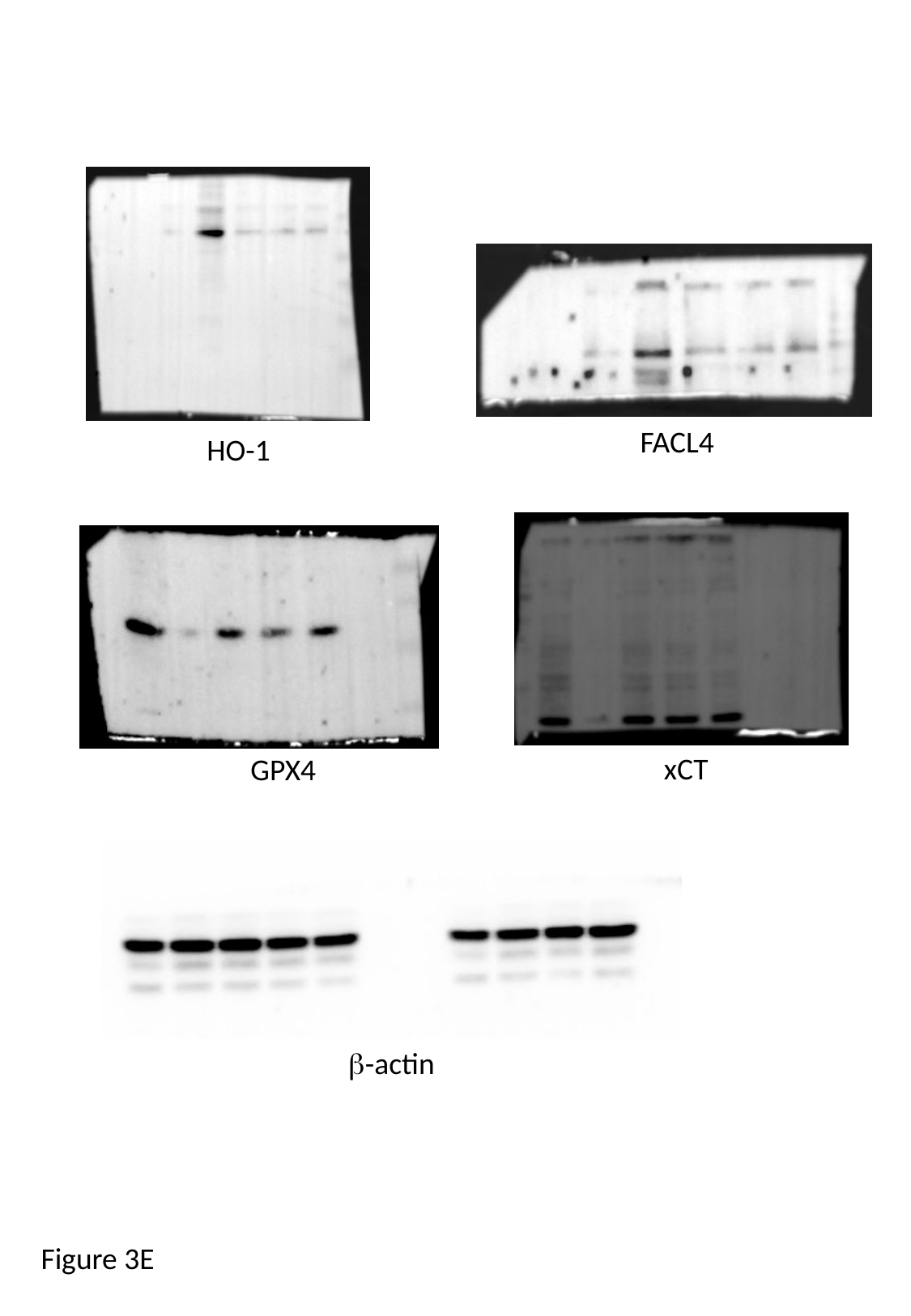

FACL4
HO-1
xCT
GPX4
β-actin
Figure 3E

## Slide 4
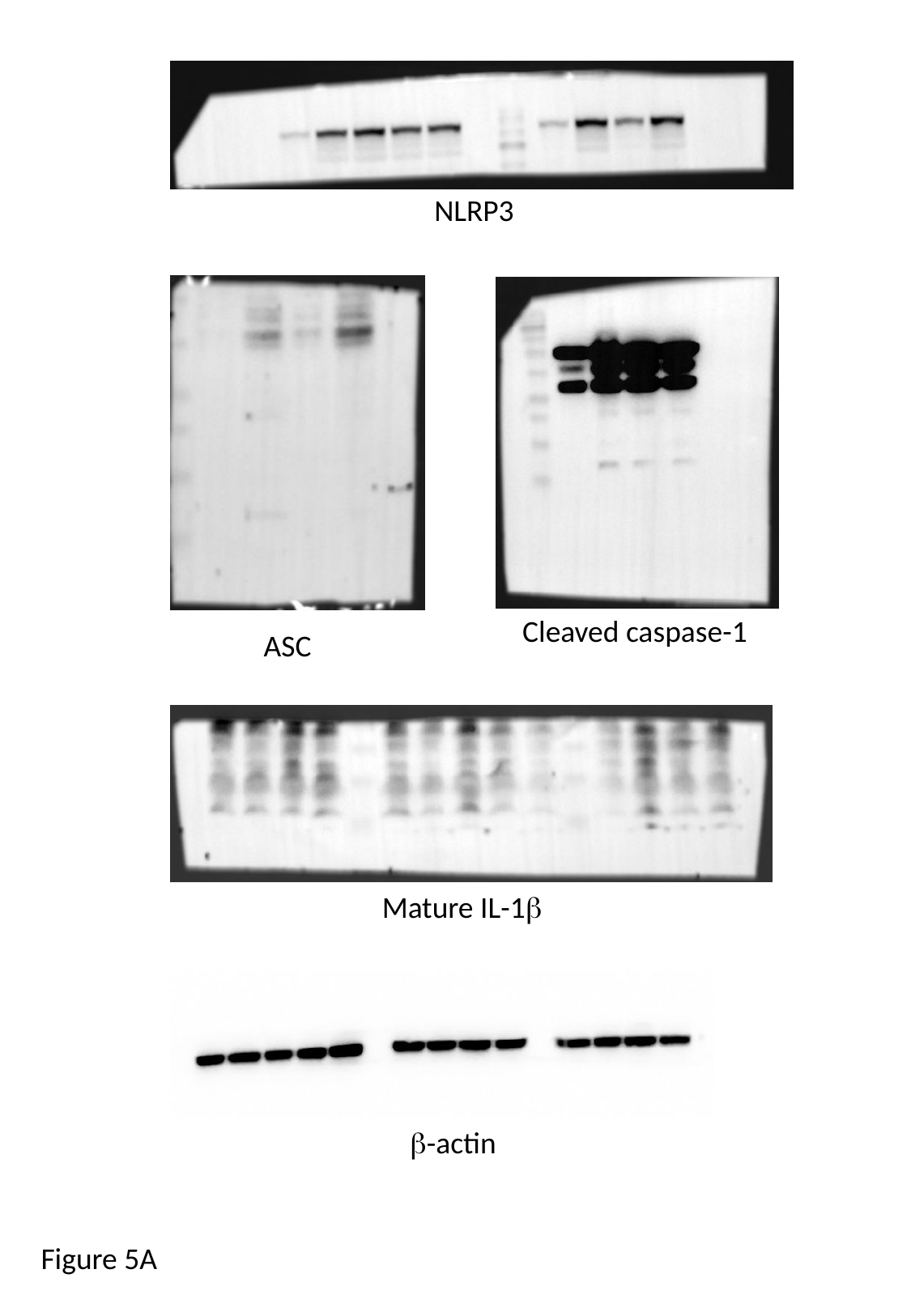

NLRP3
Cleaved caspase-1
ASC
Mature IL-1β
β-actin
Figure 5A

## Slide 5
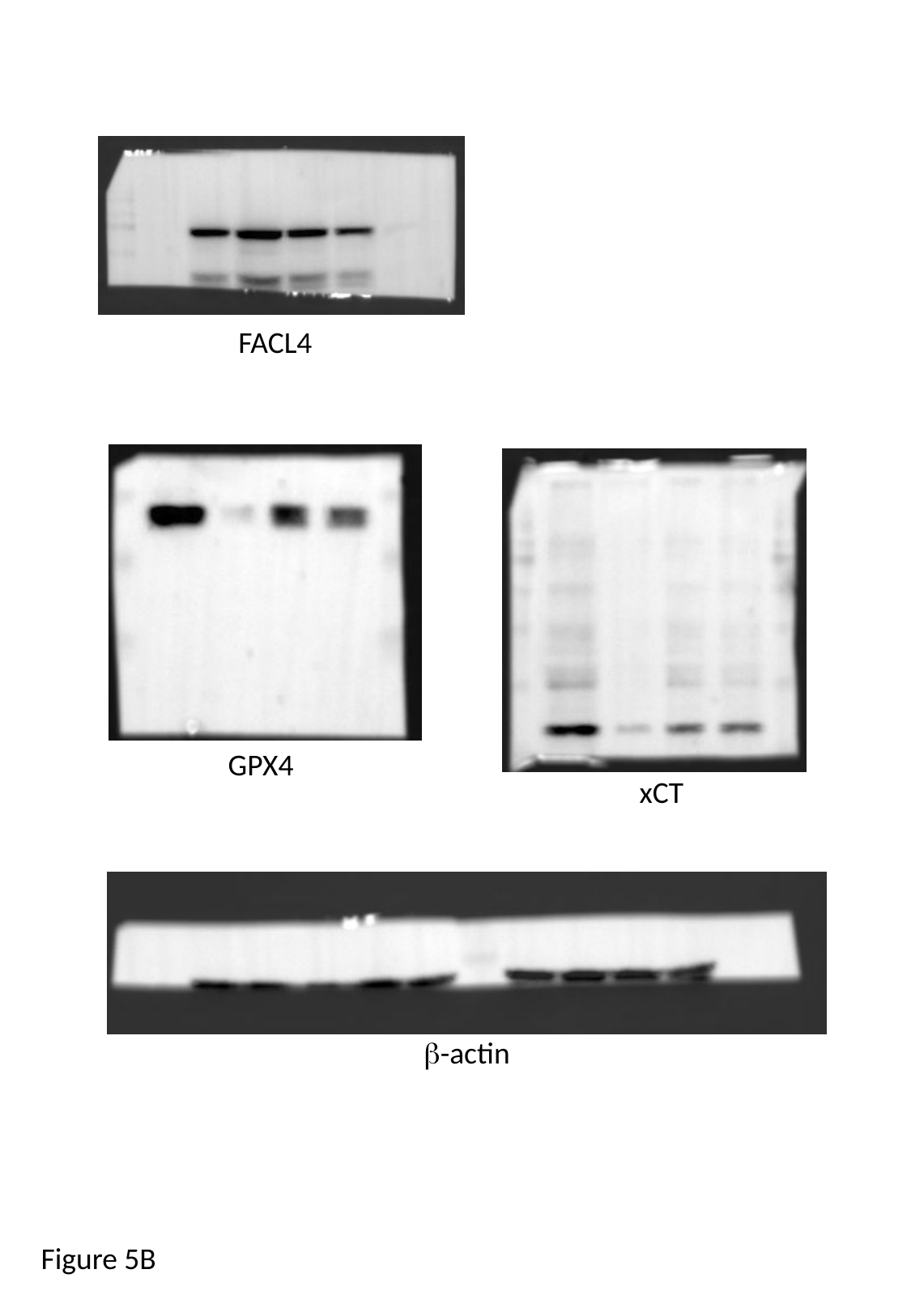

FACL4
GPX4
xCT
β-actin
Figure 5B

## Slide 6
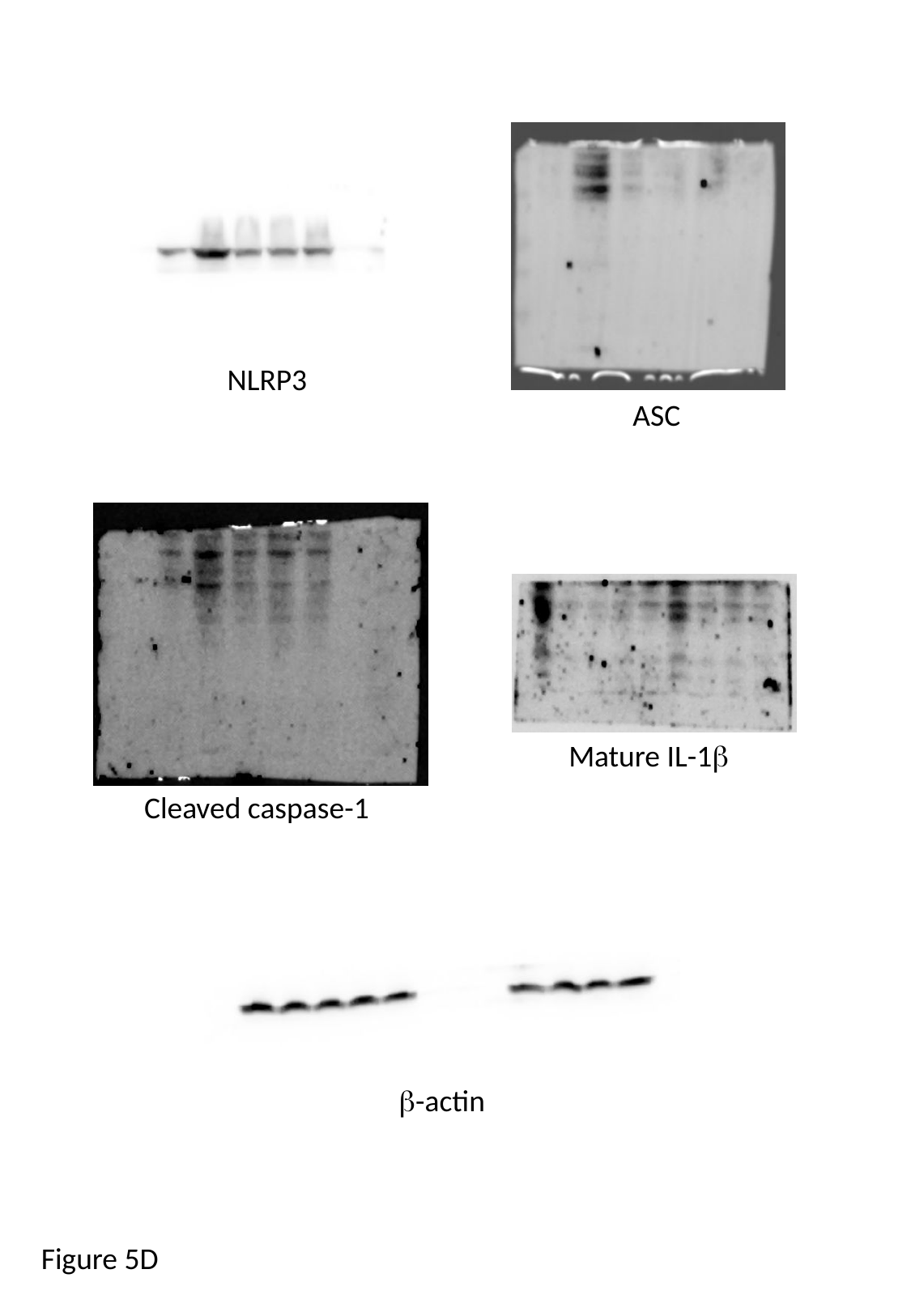

NLRP3
ASC
Mature IL-1β
Cleaved caspase-1
β-actin
Figure 5D

## Slide 7
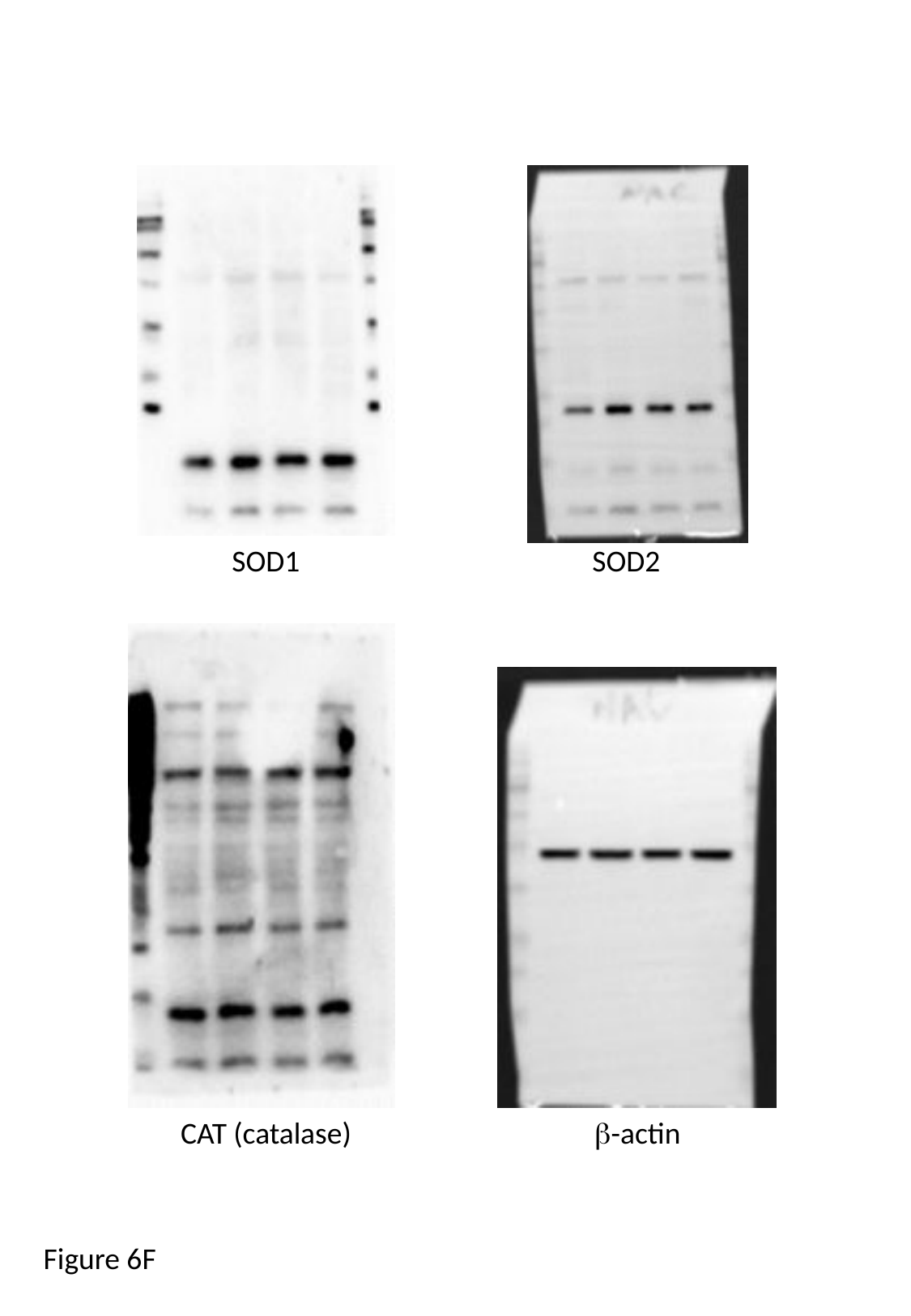

SOD1
SOD2
CAT (catalase)
β-actin
Figure 6F

## Slide 8
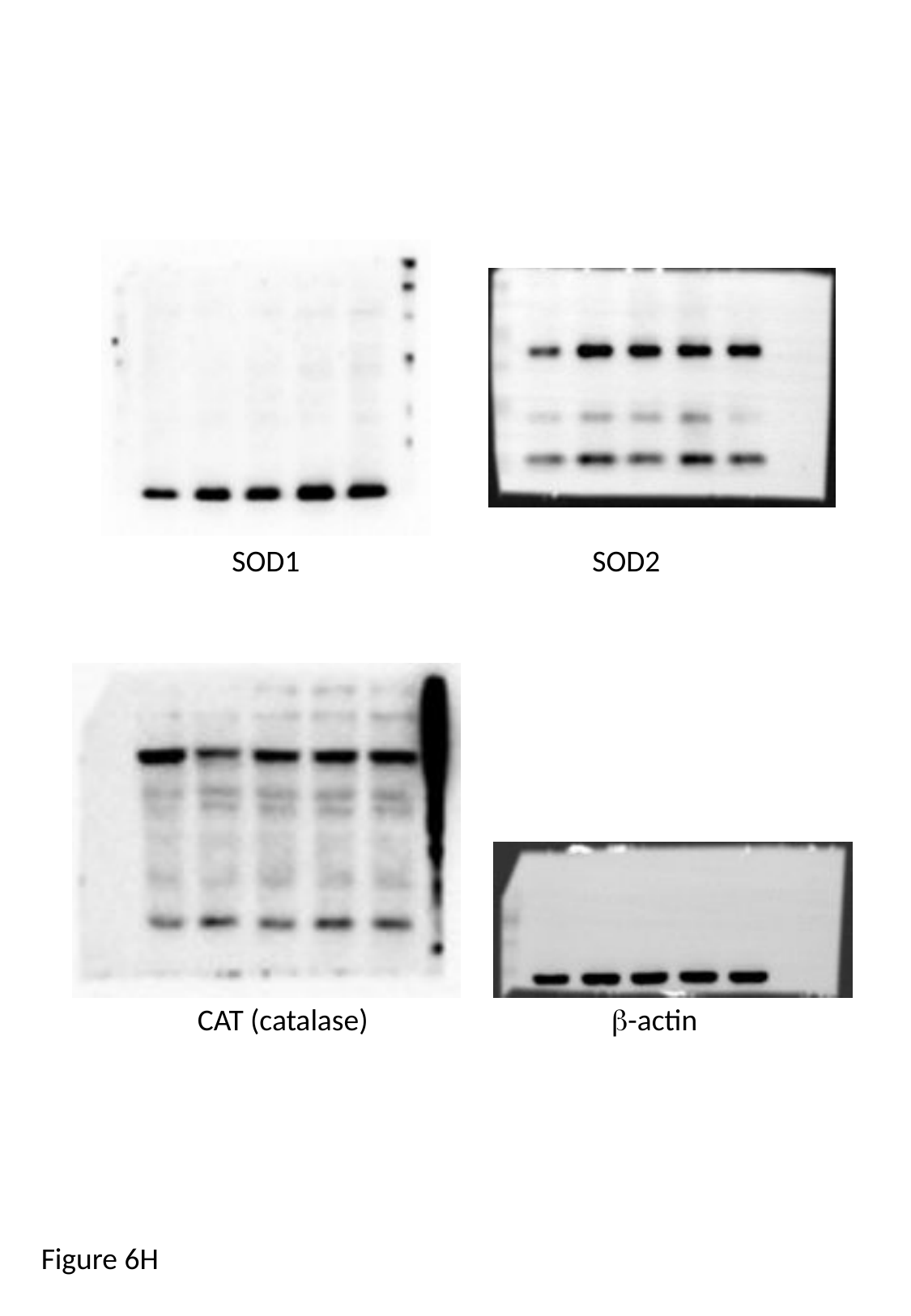

SOD1
SOD2
CAT (catalase)
β-actin
Figure 6H

## Slide 9
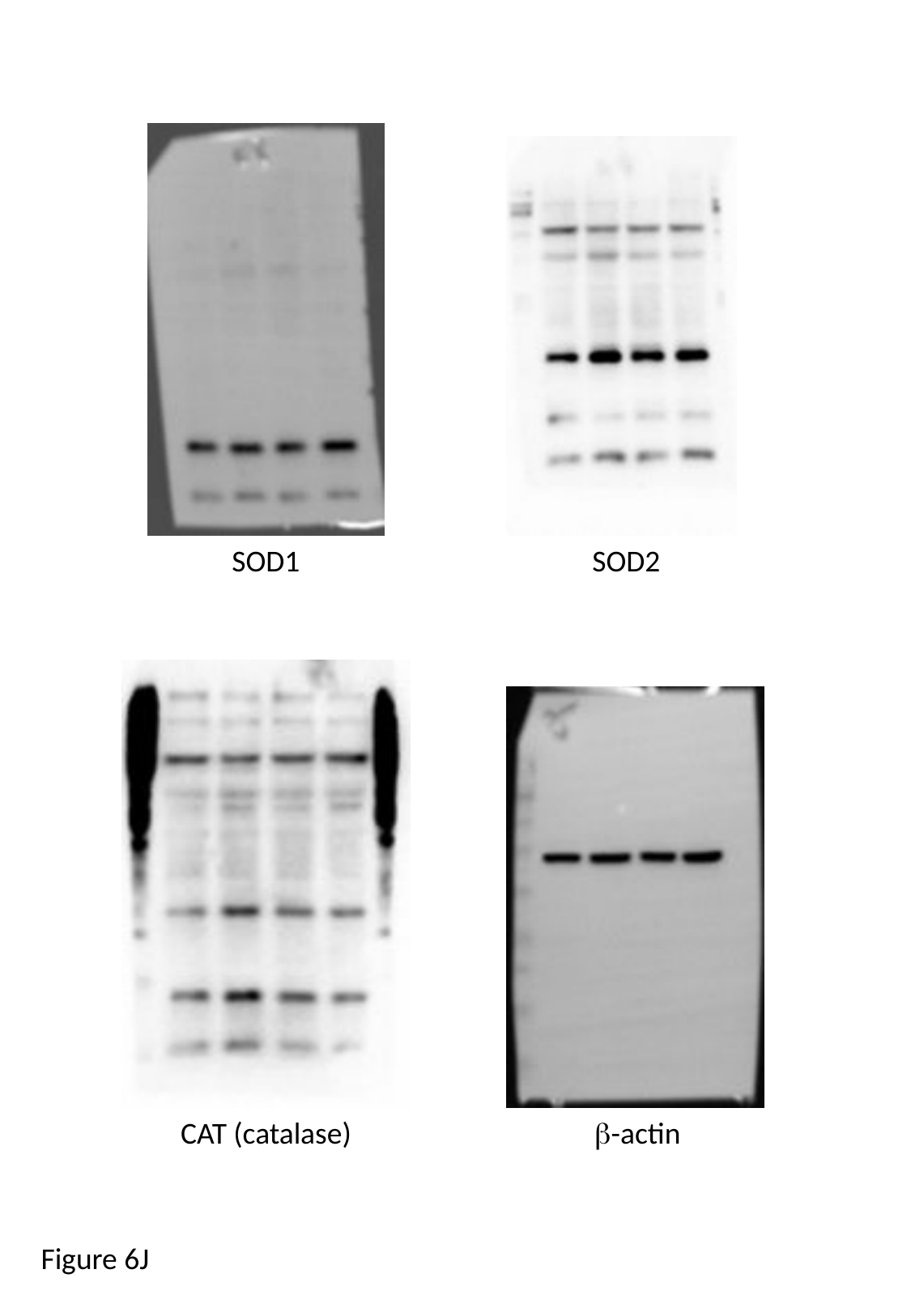

SOD1
SOD2
CAT (catalase)
β-actin
Figure 6J

## Slide 10
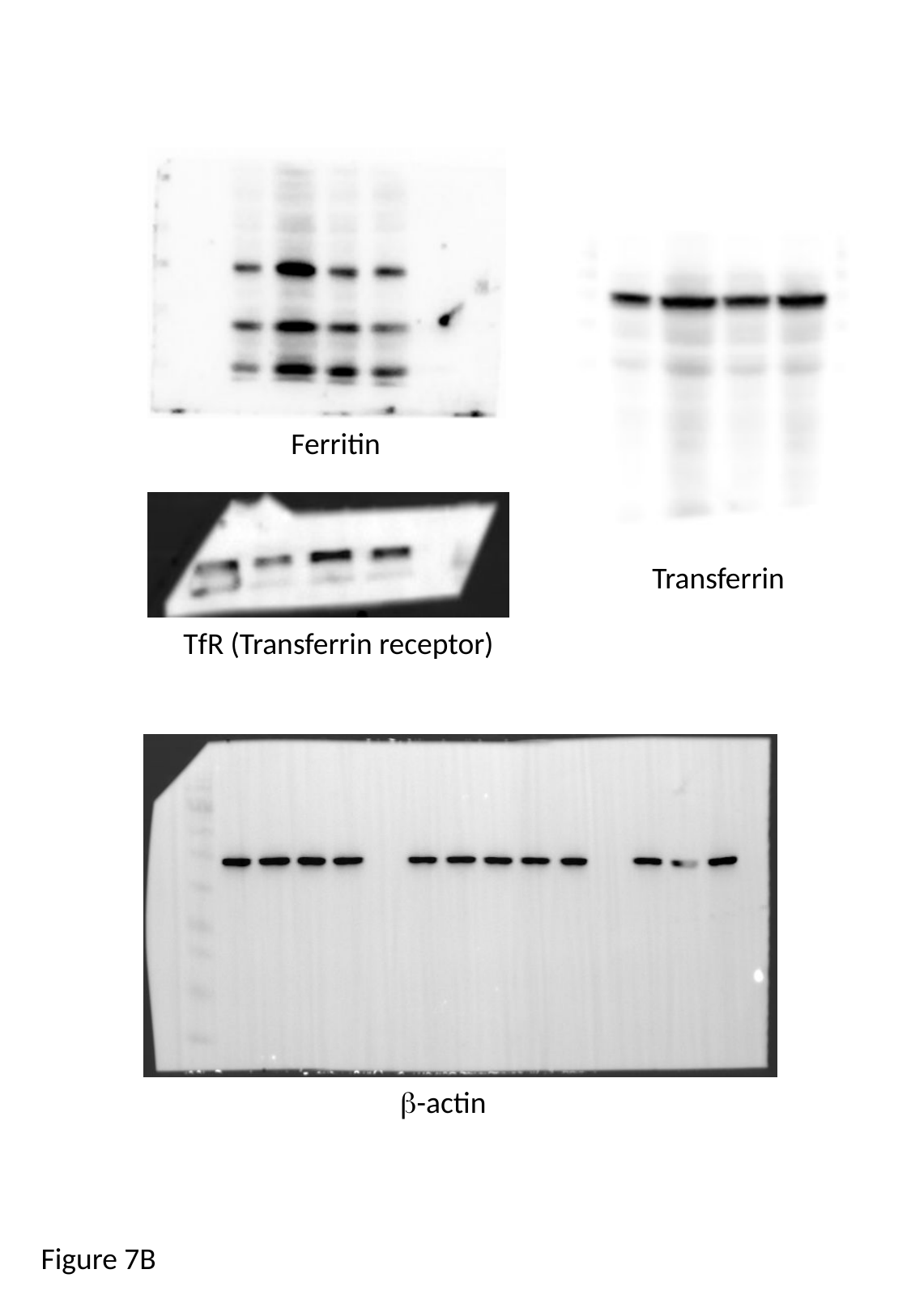

Ferritin
Transferrin
TfR (Transferrin receptor)
β-actin
Figure 7B

## Slide 11
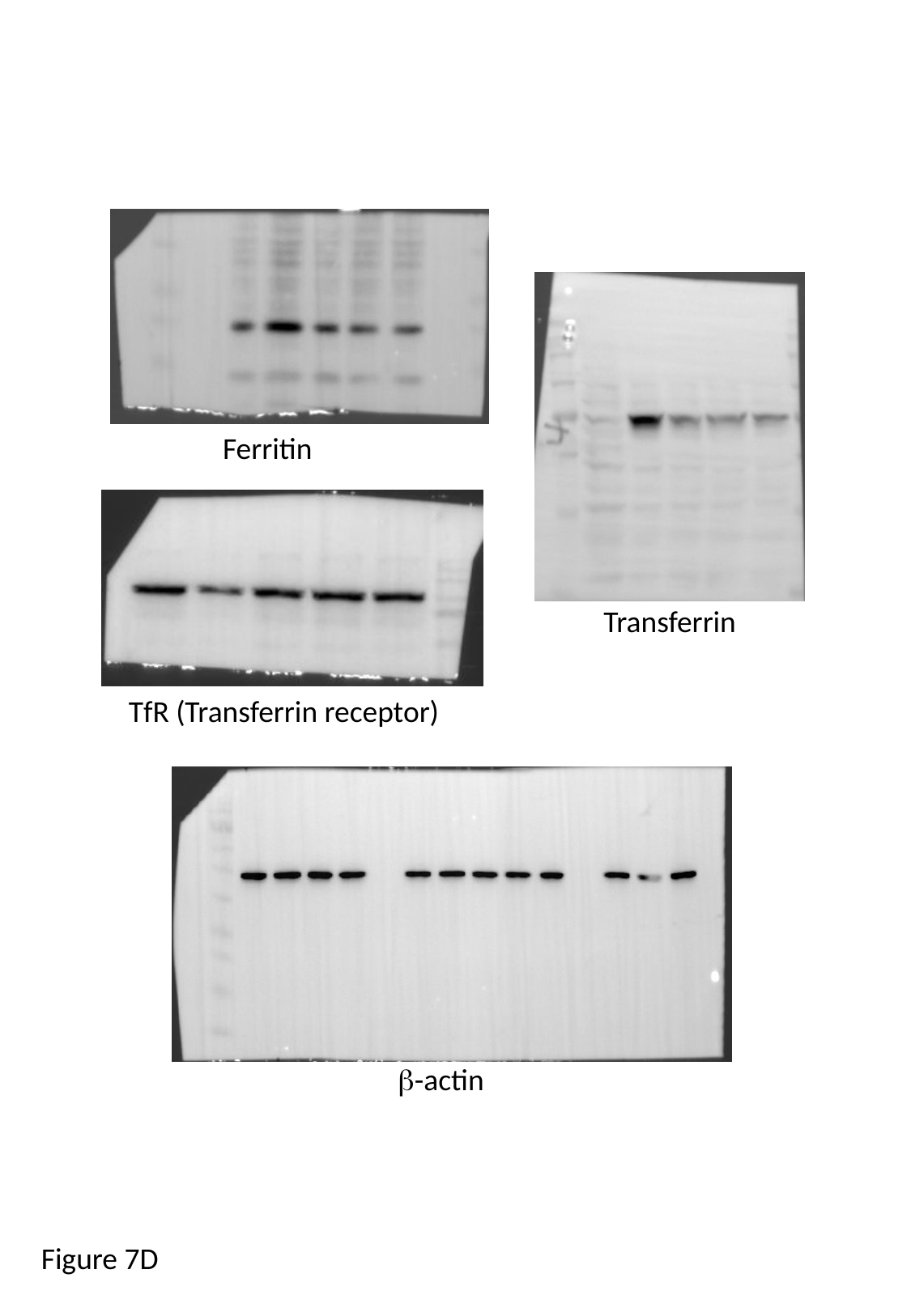

Ferritin
Transferrin
TfR (Transferrin receptor)
β-actin
Figure 7D

## Slide 12
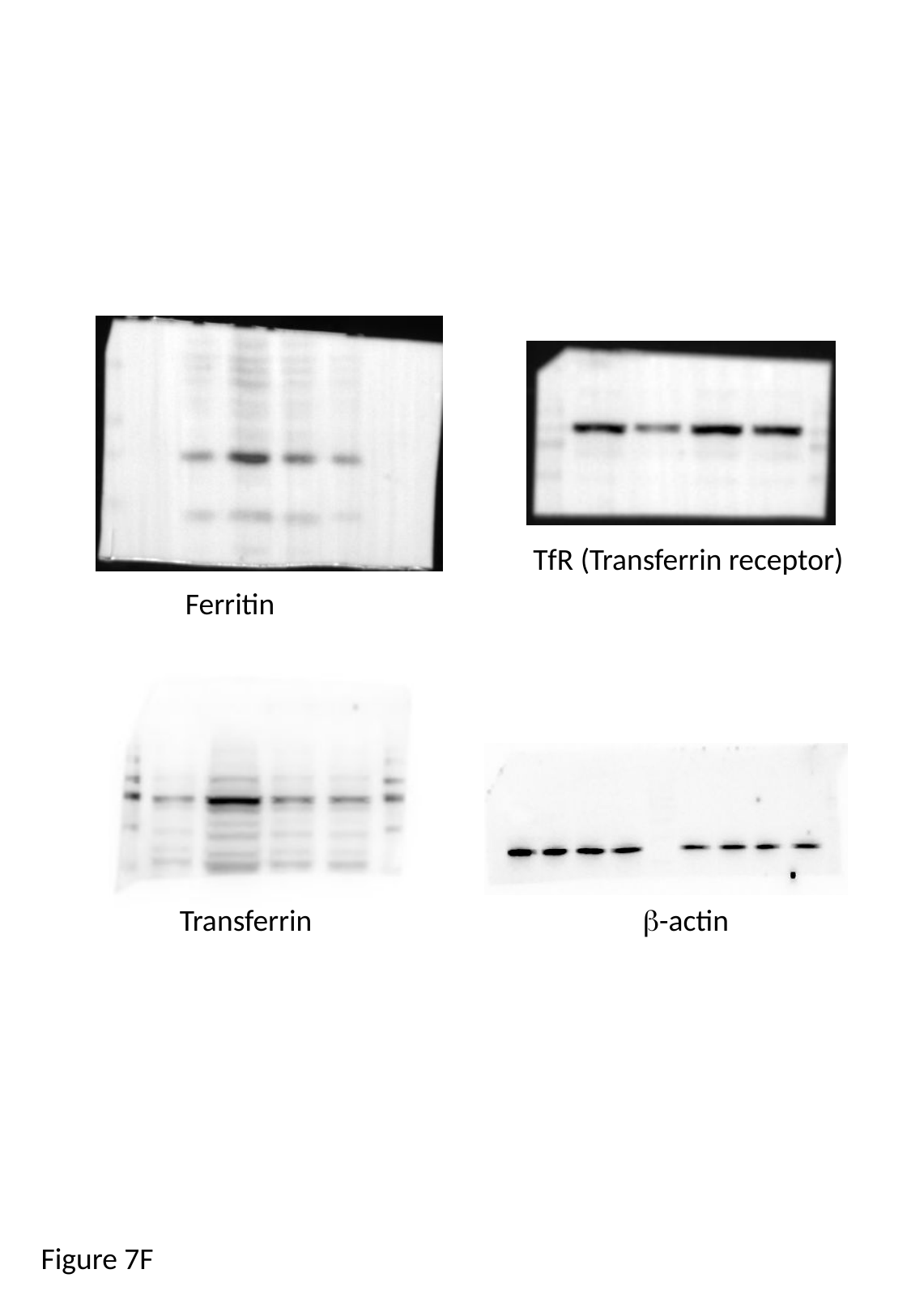

TfR (Transferrin receptor)
Ferritin
Transferrin
β-actin
Figure 7F
